# Pharmacology and Phytochemistry of Ecuadorian Medicinal Plants: An Update and Perspectives

**DOI:** 10.3390/ph14111145

**Published:** 2021-11-11

**Authors:** Chabaco Armijos, Jorge Ramírez, Melissa Salinas, Giovanni Vidari, Alírica I. Suárez

**Affiliations:** 1 Departamento de Química, Universidad Técnica Particular de Loja, Loja 1101608, Ecuador; jyramirez@utpl.edu.ec (J.R.); masalinas4@utpl.edu.ec (M.S.); alirica1@yahoo.es (A.I.S.); 2 Medical Analysis Department, Faculty of Science, Tishk International University, Erbil 44001, Iraq; 3 Facultad de Farmacia, Universidad Central de Venezuela, Caracas 1040, Venezuela

**Keywords:** medicinal plants, Ecuador, traditional uses, phytochemistry, biological/pharmacological activities, ethnobotany

## Abstract

The use of plants as therapeutic agents is part of the traditional medicine that is practiced by many indigenous communities in Ecuador. The aim of this study was to update a review published in 2016 by including the studies that were carried out in the period 2016–July 2021 on about 120 Ecuadorian medicinal plants. Relevant data on raw extracts and isolated secondary metabolites were retrieved from different databases, resulting in 104 references. They included phytochemical and pharmacological studies on several non-volatile compounds, as well as the chemical composition of essential oils (EOs). The tested biological activities are also reported. The potential of Ecuadorian plants as sources of products for practical applications in different fields, as well the perspectives of future investigations, are discussed in the last part of the review.

## 1. Introduction

The geographic location of Ecuador, together with its geological features, makes the country’s biodiversity one of the richest in the world. Ecuador is, indeed, considered among the 17 megadiverse countries, accounting for about 10% of the entire world plant species, and every year new plants are discovered and added to the long list of the species already known. This fact makes Ecuador an invaluable source of potentially new natural products of biological and pharmaceutical interest, such as carnosol, tiliroside [1], and dehydroleucodine (DL) [2]. Moreover, most plants are considered to be medicinal, where they are a fundamental part of the health systems of several Ecuadorian ethnic groups [3]. The knowledge of traditional healer practitioners has been maintained over hundreds or even thousands of years [4]. Therefore, herbal remedies have gained acceptance thanks to the apparent efficacy and safety of plants over the centuries [5]. As a result, several doctors, especially in government intercultural health districts, practice integrated forms of modern and traditional medicine nowadays.

Scientific evidence of the therapeutic efficacy and absence of toxicity in Ecuadorian medicinal plants and their products has started to be collected only in the last few decades by the researchers of several groups in different Ecuadorian Universities. This scientific activity has increased dramatically in recent years, thanks to the support of the Ecuadorian people and government authorities, who consider the sustainable use of biodiversity resources a possible source of economic wealth.

This review gives a comprehensive analysis of recent phytochemical and biologically oriented studies that were carried out on Ecuadorian medicinal plants and is focused on the potential relationships between traditional uses and pharmacological effects, assessing the therapeutic potential of natural remedies. This review completes the information that was provided by our group in 2016 [3]. Since then, more than 100 scientific articles have been published concerning phytochemical and pharmacological studies of more than 120 plants belonging to 42 different botanical families. In addition, a few naturally derived products have been patented [6]. Moreover, traditional natural preparations, such as *Colada morada*, which is consumed on the Day of the Dead (*Día de los Muertos*) [7], and *Horchata lojana*, which is a typical beverage that is made of a mixture of medicinal and aromatic plants consumed by the people of southern Ecuador [8,9,10], have received great attention. Other typical preparations are an infusion of *guaviduca* from *Piper carpunya* Ruiz & Pav. [11], which is a traditional drink of the Amazonian people, and the infusion of *Ilex guayusa* Loes., which is an emblematic tree of the Amazon Region of Ecuador that is widely used in folk medicine, ritual ceremonies, and for making industrial beverages [12,13].

**Figure 1:**
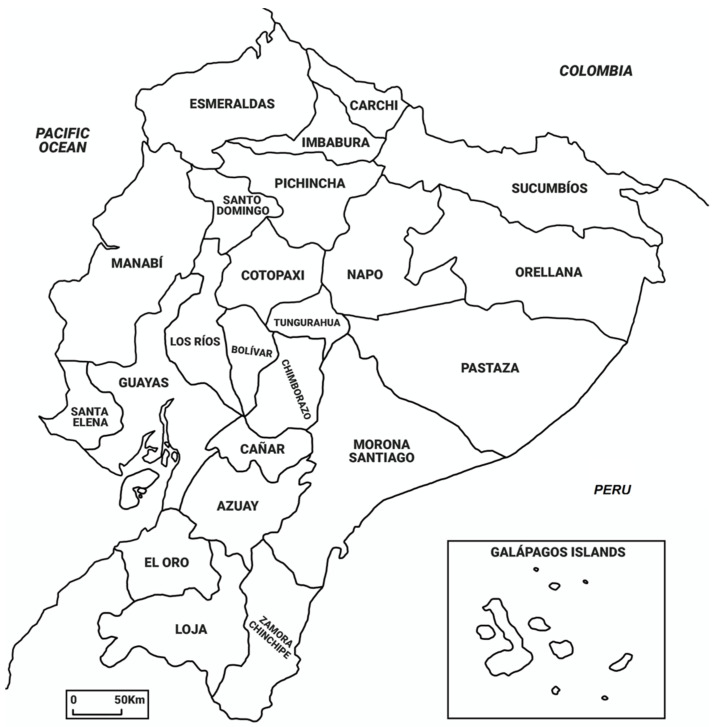
Provinces of Ecuador.

Many of the scientific articles mentioned in this review refer to studies that were carried out on plants and traditional preparations from southern Ecuador, especially from the province of Loja (Figure 1), which has a long tradition in exporting medicinal plants of great importance for human health, such as quina (*Cinchona* spp.) and condurango (*Marsdenia condurango* Rchb.f.).

Possible future research directions are also discussed in this review. In addition, the therapeutic potential of some herbal products for the development of new drugs was indicated.

## 2. Literature Search Strategies and Sources

Relevant data on medicinal plants from Ecuador were retrieved using the keywords “medicinal plants from Ecuador,” “pharmacology,” “toxicity,” “phytochemistry,” and “biological studies” in different databases, including Pubmed, SciFinder, Springer, Elsevier, Wiley, Web of Science, and Google Scholar. The search range was 2016–July 2021. The plant names and authorities were checked with the database WFO (2021): World Flora Online, published on the Internet at http://www.worldfloraonline.org (accessed on 25 September 2021). Data contained in Doctorate and Master’s theses were not considered. Articles on specific studies of Andean or Amazonian foods and fruits were not analyzed.

## 3. Phytochemical and Biological Activity Data

**Figure 2:**
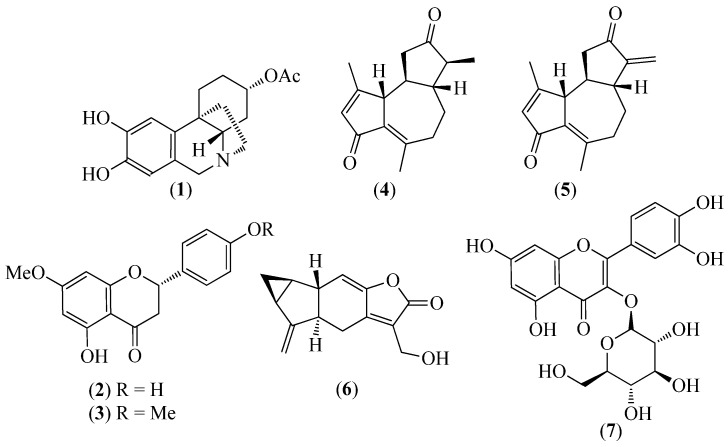
Structures of compounds **1** from *Pseudodranassa* spp., **2** and **3** from *Baccharis obtusifolia*, **4** and **5** from *Gynoxis verrucosa*, **6** from *Hedyosmum racemosum*, and **7** from *Clusia latipes*.

**Figure 3:**
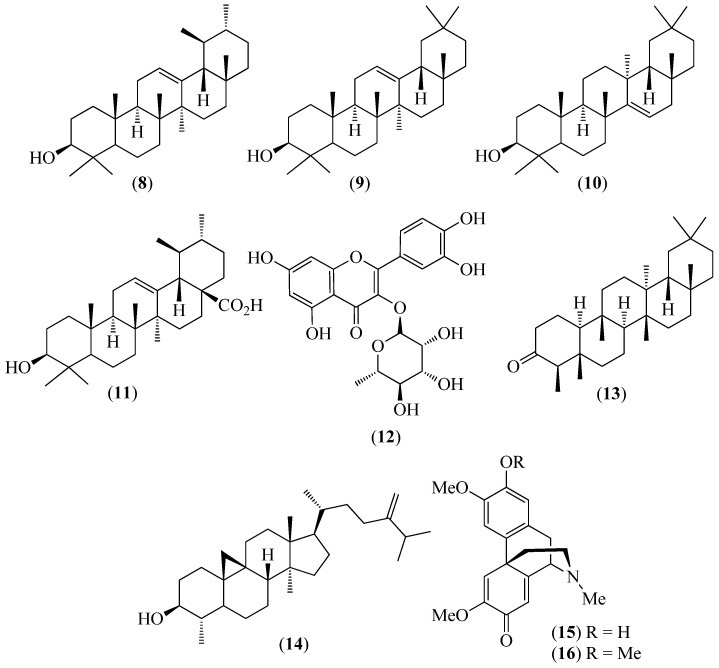
Structures of compounds **8**–**12** from *Bejaria resinosa* and **13**–**16** from *Croton ferrugineus*.

**Figure 4:**
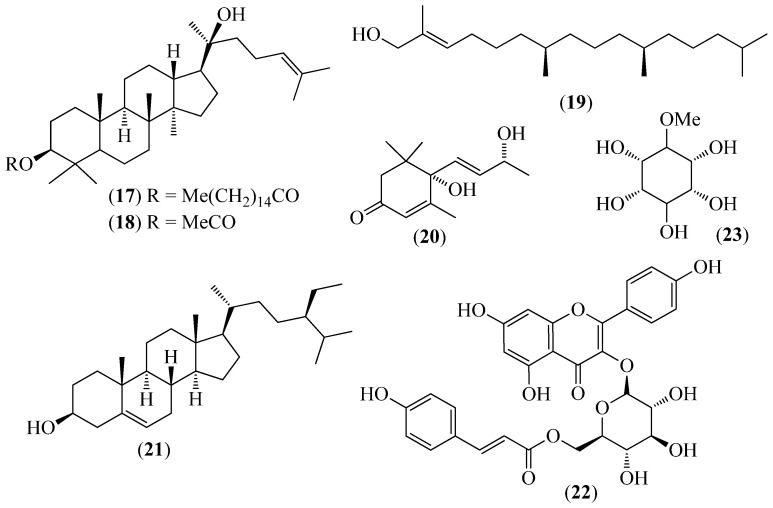
Structures of *compounds* **17**–**23** from *Croton thurifer*.

**Figure 5:**
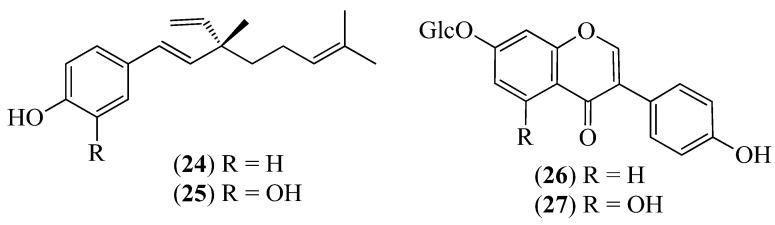
Structures of compounds **24**–**27** from *Otholobium mexicanum*.

**Figure 6:**
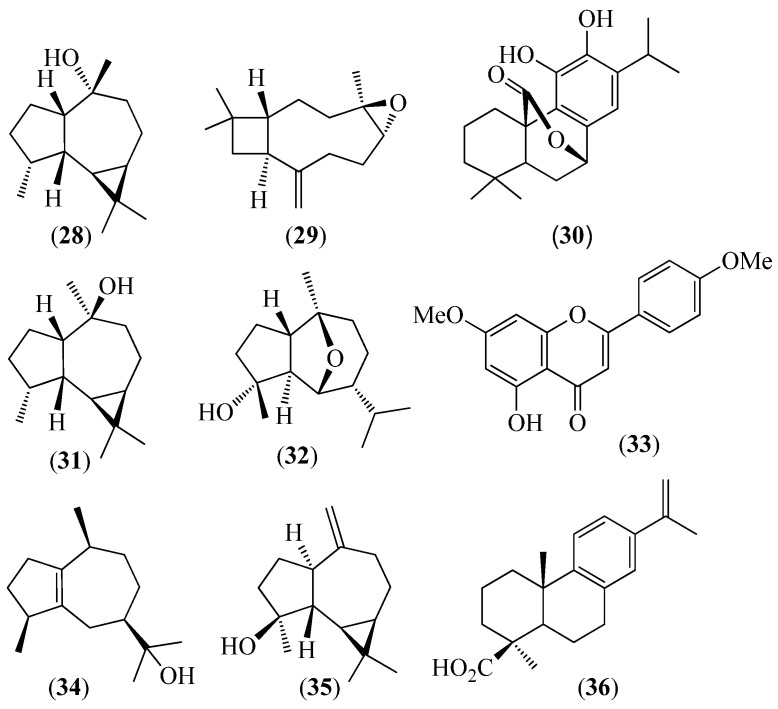
Structures of compounds **28** and **29** from *Lepechinia heteromorpha*; **30**–**33** from *L. mutica*; **28**–**30** and **34** from *L. paniculata*; and **33**, **35**, and **36** from *L. radula*.

**Figure 7:**
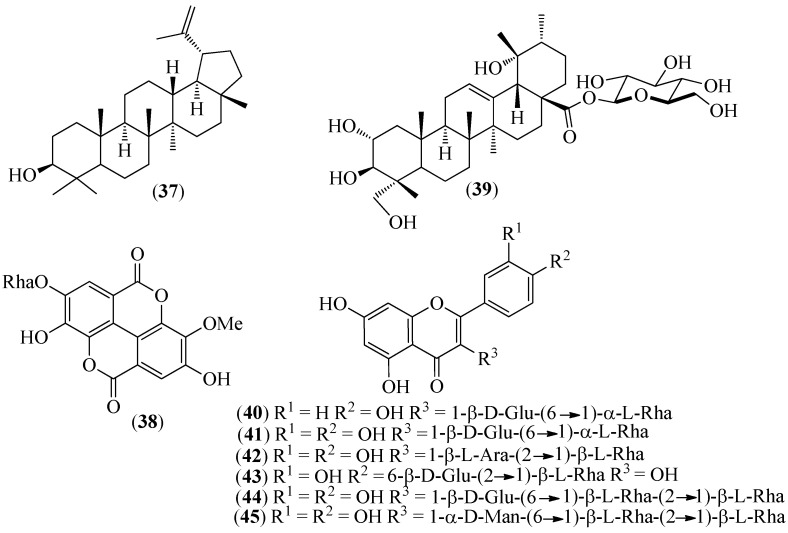
Structures of compounds **37**–**39** from *Grias neubertii* and **40**–**45** from *Gaiadendron punctatum*.

**Figure 8:**
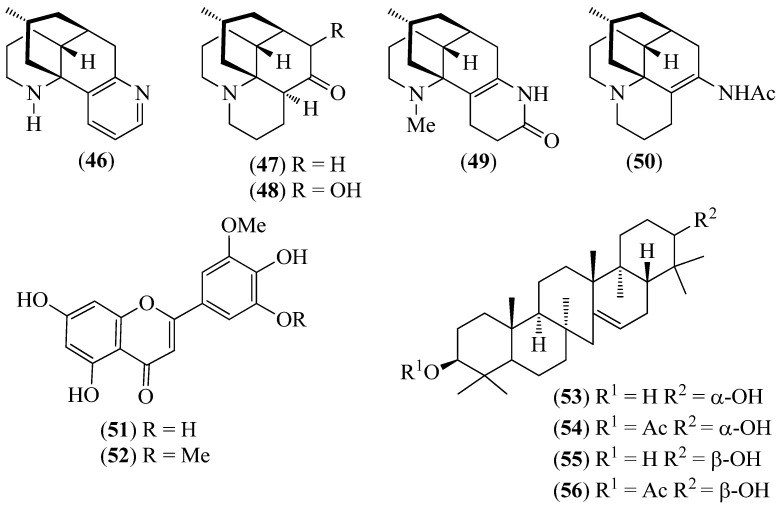
Structures of compounds **46**–**50** from *Huperzia compacta*, *H. columnaris*, and *H. tetragona*; **51** and **52** from *H. brevifolia* and *H. espinosana*; and **53**–**56** from *H. crassa*.

**Figure 9:**
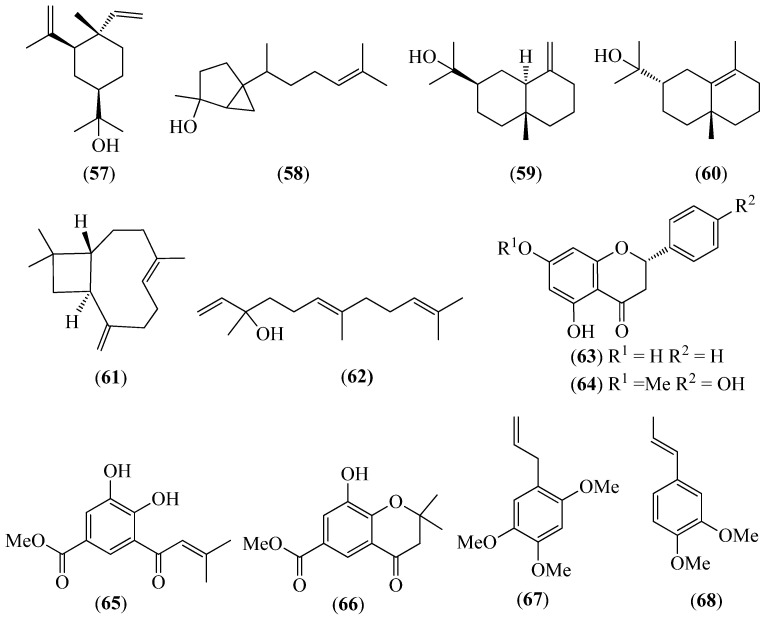
Structures of compounds **57**–**60** from *Piper barbatum*; **61** and **62** from *P. coruscans*; **62** and **63** from *P. ecuadorense*; **64**–**66** from *Piper lanceifolium*; and **61**, **62**, **67** and **68** from *P. pubinervulum*.

**Figure 10:**
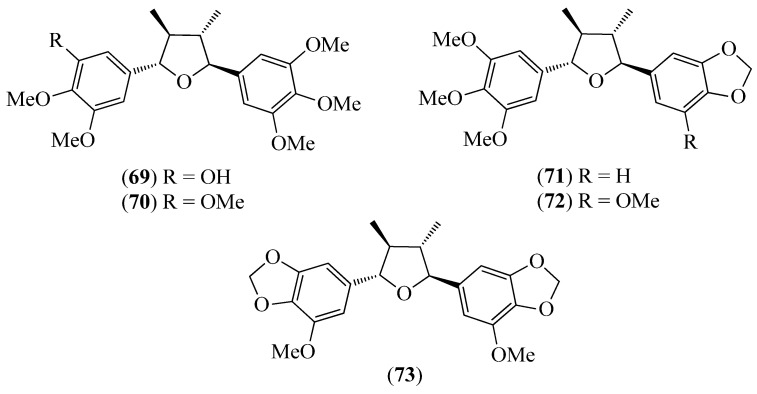
Structures of compounds **69**–**73** from *Piper subscutatum*.

**Figure 11:**
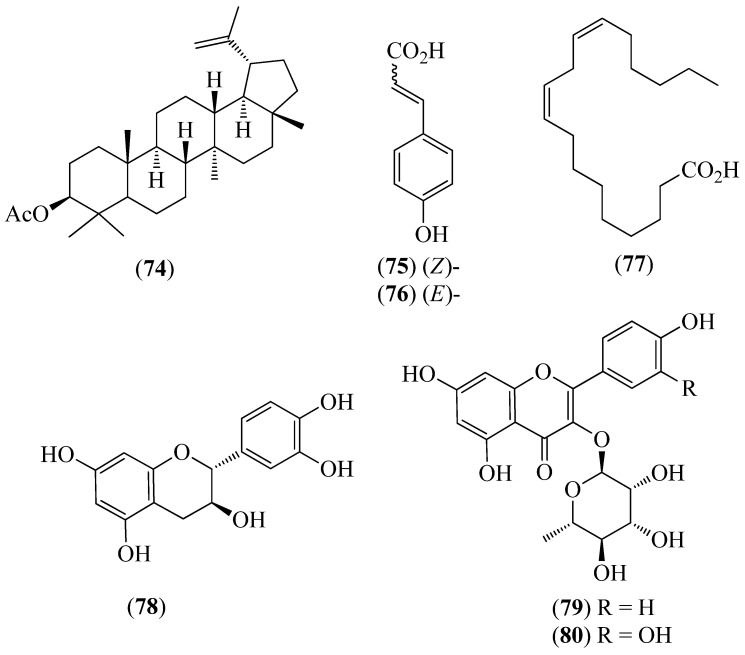
Structures of compounds **74**–**80** from *Muehlenbeckia tamnifolia*.

**Figure 12:**
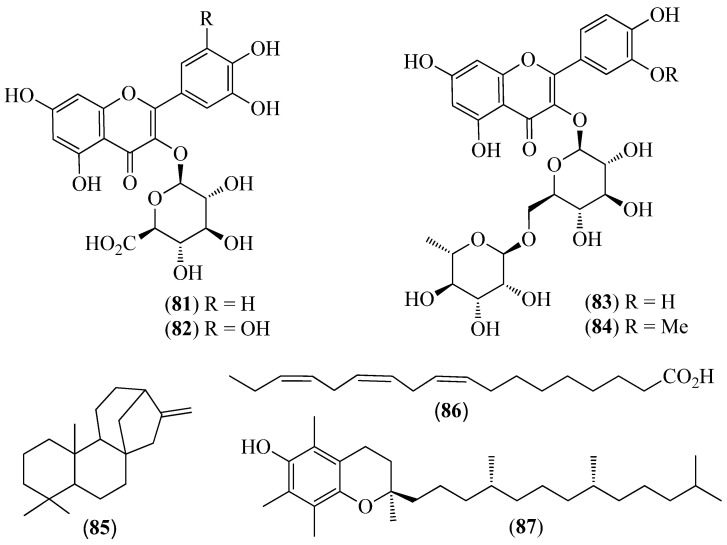
Structures of compounds **81**–**84** from *Oreocallis grandiflora* and **85**–**87** from *Roupala montana*.

**Figure 13:**
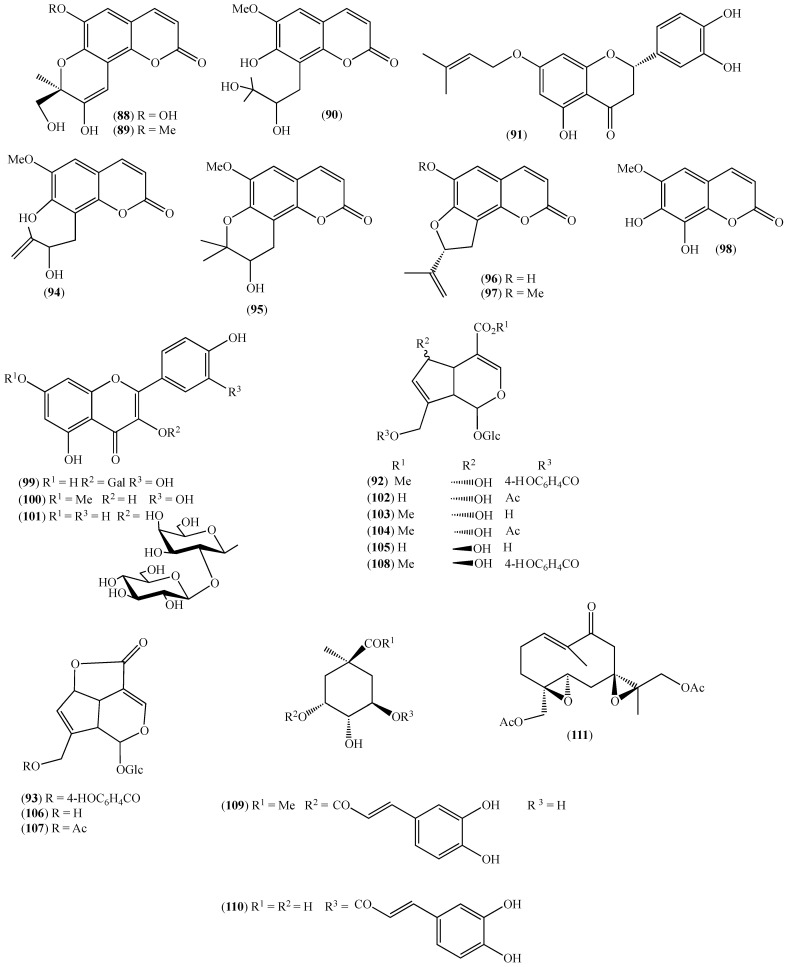
Structures of compounds **88**–**110** from *Arcytophyllum thymifolium* and **111** from *Siparuna echinata*.

The literature information is summarized in Table 1, where the plants, in alphabetical order, were grouped in their corresponding botanical family. For each species, the vernacular name and some botanical information, when available, are indicated, together with the traditional use and the phytochemical and the biological activity data when available. The structures of some characteristic compounds are reported in Figure 2, Figure 3, Figure 4, Figure 5, Figure 6, Figure 7, Figure 8, Figure 9, Figure 10, Figure 11, Figure 12 and Figure 13.

## 4. Conclusions and Perspectives

The criteria for investigating most of the 120 species cited in this review appeared to be based mainly on an ethnobotanical and ethnopharmacological approach. Indeed, scientific evidence has often confirmed traditional uses; however, not rarely, tested biological activities were not strictly related to the traditional uses. On the other hand, plants were not collected with the aim of including extracts or products in high throughput screening programs. This strategy should, instead, be involved in future research projects since it is the only investigational system that is available for discovery programs that addresses the effects of natural products on selected enzymes and receptor targets emanating from molecular biology.

Essential oils (EOs) were the most frequently investigated products. In general, oil compositions were fully determined using GC/MS and GC/FID analyses; in addition, the oil enantiomer composition and odorant characteristics were often established. As regards the biological activities of the EOs, the activity of *Renealmia thyrsoidea* EO against *Escherichia coli* and *Pseudomonas aeruginosa* [102], as well as the antifungal activity of *Lepechinia radula* [63], *Ocimum campechianum* [65], *Piper ecuadorense* [88], and *Piper pubinervulum* [90] EOs against *Candida* and *Trichophyton* strains, which are common causes of severe forms of candidiasis and dermatophytosis, are of great interest. Moreover, it is important to underline the strong acaricidal activity of a mixture of *Bursera graveolens* and *Schinus molle* EOs [44], the repellent effects of *Dacryodes peruviana* EO against mosquitoes [45], and the anti-termite properties of *Ocotea quixos* EO [70].

Thus, many EOs have the potential to be used not only as components of new perfumes due to the pleasing organoleptic properties but also as ingredients in the formulations of phytocosmetics, as well as antiseptic and insect repellent products. Moreover, essential oils should be screened in the future against clinically important bacteria and strains that are resistant to common antibiotics.

Alzheimer’s disease is the most common cause of dementia affecting elderly people and it is associated with a loss of cholinergic neurons in parts of the brain. Cholinesterase inhibitors (ChEIs) delay the breakdown of acylcholine that is released into synaptic clefts and so enhance cholinergic neurotransmission; thanks to these effects, ChEIs are considered efficacious at treating mild-to-moderate AD. In this context, the study of EO cholinesterase inhibitory activity is a relatively new area of research; in particular, the oil mechanisms of action have been poorly investigated so far. It is, therefore, of great interest that several EOs described in this review exhibited such inhibitory effects; in particular, the highly selective BuChE inhibitory activity exhibited by *Clinopodium brownei* [58], *Coreopsis triloba* [35], *Myrcianthes myrsinoides* [77], and *Salvia leucantha* [66] EOs is worthy of further studies. Equally interesting is the ChEI activity that was found for the flavonoid tiliroside, the diterpene carnosol (**30**) [1], and the alkaloids found in a few *Phaedranassa* species [17,18].

Concerning the non-volatile fractions and isolated compounds, the studies were less systematic and the compounds that are responsible for many plants’ activities are still unknown. Isolated compounds belonged to different biosynthetic families, including new ones, such as the high-molecular-weight alkaloids occurring in some *Huperzia* species, whose complete structures are, however, still unknown [4]. Extracts and isolated metabolites were subjected, almost routinely, to antiradical, e.g., DPPH, ABTS, and antioxidant (e.g., β-CLAMS and FRAP) assays. These tests are expected and, therefore, of little scientific significance for extracts containing phenolic compounds, unless high antioxidant products may be developed as phytotherapeutic agents or food supplements with health-promoting activities through the in vivo reduction of the oxidative stress. In this context, the high antioxidant activities of *Baccharis obtusifolia* [20], *Oreocallis grandiflora* [94], and *Zingiber officinale* [103] are worthy of note.

Oxidative stress induces the activation of pro-inflammatory cytokines and subsequent inflammation; therefore, the in vitro antioxidant activity of a product is often considered good evidence of its anti-inflammatory property. However, a more scientifically sound approach should require the study of the molecular mechanisms that underline anti-inflammatory activities. In this context, the expression of mitogen-activated protein kinases (MAPKs) or the release of numerous pro-inflammatory mediators, such as COX-2, the inducible nitric oxide synthase (iNOS), and interleukins IL-1β and IL-6, play a major role in the pathogenesis of various inflammatory disorders and, thus, serve as significant biomarkers for the assessment of the inflammatory process. The investigation of the anti-inflammatory effects of *Salvia sagittata* ethanolic extract [68] is a significant example of such an approach.

Several extracts and isolated compounds that were discussed in this review showed interesting inhibitory activity of the enzymes α-glucosidase and/or α-amylase. Indeed, pancreatic and intestinal glucosidases are the key enzymes of dietary carbohydrate digestion, and inhibitors of these enzymes may be effective in slowing glucose absorption to suppress postprandial hyperglycemia. In this context, it is significant to mention that the extracts and phenolic or flavonoid contents of *Gaiadendron punctatum* [73], *Muehlenbeckia tamnifolia* [93], *Oreocallis grandiflora* [19], and *Otholobium mexicanum* [56], as well as *trans*-tiliroside (**22**) [53], prenyloxy eriodictyol (**92**), and rhamnetin (**101**) [96], showed enzymatic inhibitory activity that was comparable or superior to acarbose, which is a drug that is currently used in the treatment of diabetes mellitus. Therefore, these results should promote studies on determining whether these hypoglycemic products can become sources of new antidiabetic drugs.

It is very well known that some of the most used drugs in cancer chemotherapy derive from natural products. In this context, the high antiproliferative effects shown by the extracts of some plants, such as *Annona montana* [15] and *Grias neuberthii* [72], against several human tumor cell lines are of great interest. Isolated compounds with potent in vitro cytotoxic properties include the flavonoid tricin (**52**) from *Huperzia* spp. [74], the triterpene ursolic acid (**11**) from *Bejaria resinosa* [51], and the sesquiterpene lactones onoseriolide (**6**) from *Hedyosmum racemosus* [48] and dehydroleucodine (**5**) from *Gynoxis verrucosa* [2]. The antileukemic properties of dehydroleucodine (**5**) and some derivatives were the objects of patents [6]. These findings should stimulate more systematic screening of the cytotoxic effects of Ecuadorian plants’ metabolites and the investigation of the mechanisms of the cell antiproliferative effects.

Considering the overall research activities that has been carried out so far in Ecuador on natural products, it can be concluded that little or scarce attention has been dedicated to the semi-synthesis of derivatives of isolated bioactive compounds with the aims to increase their activity, to study the structure–bioactivity relationships, and to explore the mechanisms of action and the signaling pathways that are involved in the biological activities. Even fewer efforts have been put into the synthesis of new chemical entities using computational approaches (in silico) to model the structures of natural products or to design completely new molecules. Indeed, research activities on these themes should be encouraged because they were demonstrated to be highly successful in the discovery of new bioactive compounds.

Finally, further studies, including those in vivo, are required to understand the relevance and selectivity of biological effects that have only been demonstrated in vitro so far. It is also important for practical applications to know potential acute and chronic toxicities, risks, and side effects of the plant-derived products. In fact, even raw extracts can be used as food additives and therapeutic remedies once the absence of toxicity has been demonstrated, the contents have been standardized, and the efficacy has been scientifically shown.

In conclusion, this review has clearly demonstrated the great potential of Ecuadorian plants as sources of products for different purposes and applications. Moreover, some guidelines for future research programs concerning possible sustainable uses of local therapeutic resources were indicated.

Ultimately, an important purpose of this paper is to stimulate more extensive studies on the rich medicinal flora of Ecuador.

## Figures and Tables

**Table 1:** Literature on Ecuadorian medicinal plants in the period 2016–July 2021.

Scientific Name	Common Name Botanical Data	Traditional Applications	Phytochemistry	Biological Activity
Anacardiaceae				
*Schinus molle* L.	Molle	To alleviate toothache and labor pain; as an anti-inflammatory, antiviral, antiseptic, antifungal, analgesic remedy; and to treat a supernatural ailment known by native people as “*bad air*.”	The essential oil (EO) was rich in *p*-cymene (40.0%), limonene (19.5%), myrcene (7.7%), and camphene (5.6%) [14].	The EO exhibited an important acaricidal effect: at a concentration of 2%, it was lethal to *R. sanguineus* larvae [14].
Annonaceae				
*Annona montana* M.	Guanabana de monte or guanabana (false graviola)	The plant is used to treat lice, influenza, and insomnia; immature fruits are used to treat dysentery.		Organic extracts showed genotoxic damage to DNA and cell antiproliferative activity against several human tumor cell lines. The highest activity was observed for the ethyl acetate extract, with the following percentages of inhibition: lung cancer A549 (97 ± 1.7%), cerebral astrocytoma D384 (98 ± 3.2%), breast carcinoma MCF-7 (93 ± 4.5%), prostate cancer PC-3 (99 ± 6.8%), and colon cancer RKO (96 ± 5.7%). The IC_50_ values were 9.6, 7.9, 6.0, 13.1, and 7.7 μg/mL, respectively, for each tumor cell line [15].
Amarryllidaceae				
*Crinum* × *amabile* Donn	Lirio de araña púrpura (purple spider lily) or lirio de araña gigante rosada (pink giant spider lily) in the Ecuador continent and lirio de cinta in the Galapagos Islands	It is used in the Esmeraldas province to treat hemorrhoids.	Alkaloids, flavonoids, glycosides, and carbohydrates were determined as the main compounds in leaves and bulbs using phytochemical screening [16].	The anti-inflammatory and cytotoxic activities were evaluated using isolated neutrophils and the tetrazolium salt method (WST-1), respectively [16].
*Phaendranassa brevifolia* Meerow *Phaendranassa cinerea* Ravenna *Phaendranassa cuencana* Minga, C. Ulloa & Oleas *Phaendranassa dubia* (Kunth) J.F.Macbr. *Phaendranassa glauciflora* Meerow *Phaendranassa tungurague* Ravena	Ashpa onion in Kichwa (fake onion), or papa de lobo in Spanish (fox’s potato)		The alkaloid profile of each species was analyzed using GC/MS [17,18]; a high content of lycorine-type alkaloids was found in *P. dubia* and *P. brevifolia* bulbs [18].	The in vitro inhibitory activity of *P. cinerea*, *P. cuencana*, *P. dubia*, *P. glauciflora*, and *P. tungurague* was evaluated against the enzymes acetylcholinesterase (AChE) and butyrylcholinesterase (BuChE), which are considered important targets in the mechanism of Alzheimer’s disease. Docking studies indicated that cantabricine (**1**) (Figure 2), which is an alkaloid present in most species under investigation, was active on both enzymes. However, this compound was not present in the extracts of the two plants that exhibited the highest in vitro inhibitory activity, namely, *P. cuencana* (IC_50_ = 0.88 ± 0.11 µg/mL) against AChE and *P. dubia* (IC_50_ = 14.26 ± 2.71 µg/mL) against BuChE; instead, the two species were rich in galanthamine-type alkaloids [17]. The alkaloid profiles of *P. dubia* and *P. brevifolia* bulbs were investigated and significant inhibitory activities on both enzymes were determined [18]. The IC_50_ values of the activity against AChE were 25.48 ± 0.39 and 3.45 ± 0.29 μg/mL, respectively, whereas the IC_50_ values against BuChE were 96 ± 4.94 and 58.89 ± 0.55 μg/mL, respectively. A high content of lycorine-type alkaloids was found in both species [18].
Apiaceae				
*Neonelsonia acuminata* (Benth) J. M. Coult & Rose	Zanahoria blanca (white carrot)	To relieve stomach pain.		The potential antidiabetic properties were evaluated by measuring the α-amylase and α-glucosidase inhibitory activities. No activity was reported on α-amylase, whereas the percentages of α-glucosidase inhibition were significant only at the high concentrations of 500 and 1000 µg/mL, with values of 73.02 ± 0.2 and 91.0 ± 1.0%, respectively. The IC_50_ values (µg/mL) of the antiradical and antioxidant activities, as determined using 2,2-diphenyl-1-picrylhydrazyl (DPPH), showed that the Trolox (a water-soluble derivative of vitamin E) equivalent antioxidant capacity (TEAC) and the β-carotene-linoleic acid model system (β-CLAMS) assays were 31.6 ± 0.2, 23.0 ± 1.93, and 43.1 ± 0.81, respectively [19].
Apocynaceae				
*Prestonia mollis* Kunth	Bejuco de cancer	Anticancer, wound healing, and disinfectant remedy.		The plant showed no significant antioxidant and antiradical effects [20].
*Prestonia* spp.	Chicle (gum)	To treat skin affections.	Alkaloids, saponins, and flavonoids were identified as the main phytochemicals [21].	
Aquifoliaceae				
*Ilex guayusa* Loes.	Guayusa	Widely used in the Amazon region of Ecuador in ritual ceremonies; moreover, the traditional use for treating various ailments and diseases was well documented by several recent reviews [22].	The content of polyphenols, alkaloids, and the health benefits of a tea drink prepared with guayusa leaves together with beverages from other *Ilex* species was discussed [23]. The optimization of the ultrasound-assisted extraction of phenolic antioxidants from the leaves using response surface methodology was described [24].	Strong inhibition of the enzyme hyaluronidase indicated an interesting anti-inflammatory activity; however, no antibacterial effects were observed from *Staphylococcus aureus* and *Escherichia coli* [25]. In an attempt to validate the traditional use of guayusa and alfalfa for the treatment of female infertility, the estradiol levels in blood samples were measured after administration of the plants to rats, which showed high in vivo estrogenic effects [26]. A methanolic extract of guayusa exerted a significant inhibitory effect on α-glucosidase (IC_50_ value = 176.5 ± 1.50 μg/mL), while it was inactive against α-amylase. In antiradical and antioxidant assays, the IC_50_ values (μg/mL) were 14.2 ± 0.99, 11.8 ± 1.01, and 13.0 ± 0.85 in DPPH, TEAC, and β-CLAMS assays, respectively.
Arialaceae				
*Oreopanax andreanus* Marchal	Pumamaqui or manos de puma in Spanish (puma paws)	Wound healing, disinfectant, astringent, and antiseptic remedy; together with *Oreopanax eriocephalus* Harms (traditional called maqui maqui), it is employed as an anti-inflammatory and antibacterial remedy [20].		In in vitro antioxidant assays, the values of Trolox equivalents per mg extract (μM TE g^−1^) were 108.9 ± 24.9 (ABTS) and 75.7 ± 34.5 (DPPH) for *O. andreanus*, and 473.2 ± 4.7 (ABTS) and 665.6 ± 42.9 (DPPH) for *O. eriocephalus* [20].
*Oreopanax ecuadoriensis* Seem.	Pumamaky (puma paws)	Quichua people use it as a sacred plant in rituals to protect and purify people. Traditional medical treatments also include its use in postpartum baths and to cure flu and headaches.	Thirty-three compounds were detected in the leaf EO using GC/MS; the most abundant components were α-thujene (36.63%), bicyclogermacrene (8.76%), β-pinene (8.32%), and limonene (5.15%) [27].	At a concentration of 1.25% in DMSO, the average halos of growth inhibition (mm) that were shown by the EO were 6.0 ± 0.0 for *Candida albicans*, indicating no activity, 6.14 ± 0.09 for *Trichophyton mentagrophytes*, 8.84 ± 1.26 for *T. rubrum*, and 10.97 ± 0.57 for *Microsporum canis*, indicating interesting antifungal activity [27].
Araceae				
*Dieffenbachia costata* Klotzsch ex Schott *Xanthosoma purpuratum* K. Krause (syn. *X. hylaeae* Engl. & K. Krause)	Lalu Shungu panga			Aqueous extracts of both plants showed no relevant insecticidal activity against *Plutella xylostella* L. (diamondback moth) under laboratory and open-field conditions, and against *Brevicoryne brassicae* L. under semifield conditions [28].
Arecaceae				
*Mauritia flexuosa* L.f.	The popular name of the tree varies depending on the region; in Ecuador, it is known by the names canagucho and morete	The edible fruits are widely consumed in the palm-growing regions.	The content of flavones, flavonols, flavanones, phenolic acids, and carotenoids in extracts of fruits collected at three different altitudes were compared using HPLC/MS. Most flavonoid glycosides [29] contained quercetin as the aglycone.	A high antioxidant capacity, as well a good protective effect against lipid oxidation were determined, and a direct correlation between the concentration of bioactive compounds and antioxidant capacity was found [29].
Asteraceae				
*Ageratina dendroides* (Spreng.) R.M. King & H. Rob.	Pega chilca	To treat blows and wounds	The main components of the EO were identified as being germacrene D (29.92 ± 0.68%), δ-cadinene (9.31 ± 0.11%), and *cis*-cadina-1,4-diene (5.48 ± 0.04%) using GC/MS [30].	
*Baccharis genistelloides* (Lam.) Pers.	Tres filos	It is used in slimming treatments; to stimulate diuresis; and to cure stomach pain, influenza, kidney problems, and diabetes.		A methanolic extract was inactive against α-amylase, while it showed an IC_50_ = 154.6 ± 1.28 mg/mL toward α-glucosidase, which is a value that was much better than the positive control acarbose (IC_50_ = 964.6 ± 2.80 mg/mL). Regarding the radical-scavenging and antioxidant activities of the methanolic extract of the plant, the values were 98 ± 1.4 µg/mL (DPPH), 100 ± 1.0 µg/mL (TEAC), and 43 ± 0.5 µg/mL (β-CLAMS) at the concentrations of 50, 10, and 5 mg/mL, respectively. The values were comparable with those of the reference compounds α-tocopherol and Trolox [19].
*Baccharis obtusifolia* Kunth	Chilca redonda	To treat ulcers, rheumatism, liver diseases, and heal wounds.	5,4’-Dihydroxy-7-methoxyflavone (**2**) and 5-hydroxy-7,4’dimethoxyflavone (**3**) (Figure 2) were isolated and identified from the methanolic extract [31].	The flavones **2** and **3** were considered to be responsible for the cytotoxic effects (MTS assay) on prostate (PC-3), colon (RKO), astrocytoma (D-384), and breast (MCF-7) cells, especially on colon cells [31]. The antioxidant activity of the plant, whose values (μM TE) were 789.8 ± 0.7 (ABTS) and 984.4 ± 4.5 (DPPH), was considered one of the highest in comparison with numerous plants [20].
*Chromolaena laevigata* (Lam.) R.M. King & H. Rob.	Doctorcito	Antibacterial, antifungal, analgesic, anti-inflammatory, and emmenagogue effects were attributed.	The most abundant compounds between 25 components that were identified in the EO using GC/MS were laevigatin (46.84%), germacrene D (15.38%), limonene (4.94%), bicyclogermacrene (4.14%), and α-pinene (2.85%) [32].	The antibacterial and antifungal activities were evaluated using the microdilution technique against the Gram-negative bacteria *Pseudomonas aeruginosa*, *Klebsiella pneumonia*, *Proteus vulgaris*, *Salmonella typhimurium*, and *Escherichia coli*; the Gram-positive bacteria *Enterococus fecalis* and *Staphylococcus aureus*; and the fungi *Trichophyton rubrum* and *T. mentagrophytes*. A moderate inhibitory activity was observed only toward *T. rubrum* (MIC = 125 μg/mL) and *T. mentagrophytes* (MIC = 250 μg/mL) [32].
*Chuquiraga jussieui* J.F. Gmel.	Chuquiragua	To cure kidney and liver diseases and as a diuretic.	Carotenes, vitamin C, and polyphenols were the main compounds that were identified in extracts of specimens collected at different locations [33].	High antioxidant properties of the extracts; they were higher for the leaf than for the flower extract. No quantitative data were determined [33].
*Conyza bonariensis* (L.) Cronquist	Rama negra (black branch), yerba carnicera, or canilla de venado (deer shin)	To treat rheumatism, nephritis, and gout.		Antimicrobial and antifungal activities were reported for samples of the plant collected in countries that were different from Ecuador, whereas the antioxidant activity and the chemical profile of different organic extracts, determined using GC/MS, were described for a sample that was collected in Ecuador. The EtOAc fraction from a leaf methanolic extract showed the highest DPPH radical scavenging activity (90.69 ± 3.16% at 500 μg/mL) and reducing activity of the ferric tripyridyltriazine complex (Fe^3+^-TPTZ) with a value of 2.355 mg Trolox equivalent (TE)/g dry fraction. These effects could be mainly related to the presence in the fraction of eugenol, *trans*-isoeugenol, lucenin-2, methyl salicylate, and syringic acid [34].
*Coreopsis triloba* S.F. Blake (a synonym of *C. capillacea* Kunth)	Macchia, peña nachic, or caca nachic	To treat inflammation and used in bath infusions.	GC/MS analysis of the EO was undertaken via steam distillation at the macro- and microscale using a Marcusson distillator, which gave an account of the composition and enantiomeric distribution. Twenty-nine compounds where identified, including (*E*)-β-ocimene (35.2–35.9%), β-phellandrene (24.6–25.0%), α-pinene (15.3–15.9%), myrcene (10.9–11.0%), sabinene (2.2–2.4%), (*Z*)-β-ocimene (1.5%) as the main ones [35].	The EO showed selective inhibition of the enzyme BuChE with IC_50_ = 6.8 μg/mL compared with IC_50_ = 42.2 μg/mL toward AChE [35].
*Coreopsis venusta* Kunth	Ñache leñoso (woody ñache)	To relieve inflammation to women’s bellies and during labor caused by cold.		The antioxidant capacity of a plant methanolic extract, expressed as Trolox equivalents (μM TE) per milligram of extract, was 790.7 ± 1.4 in the ABTS test and 869.8 ± 15.4 in the DPPH assay [20].
*Gnaphalium elegans* C. Presl	Oreja de burro (donkey ear) (Azuay province)	Against tumors.	Twenty-one components were identified in the EO using GC/MS analysis; γ-curcumene (55.61%), italicene (4.69%), α-cubebene (4.45%), δ-cadinene (4.28%), and α-pinene (3.57%) were the main components [36].	The antimicrobial activity of the EO was tested against the bacteria *Pseudomonas aeruginosa*, *Klebsiella pneumonia*, *Proteus vulgaris*, *Salmonella typhimurium*, *Escherichia coli*, *Enterococcus fecalis*, and *Staphylococcus aureus*, and the fungi *Trichophyton rubrum* and *T. mentagrophyte*. The oil was moderately active against *T. rubrum* (MIC = 500 μg/mL) and *T. mentagrophytes* (MIC = 1000 μg/mL) [36].
*Gynoxys verrucosa* Sch.Bip. ex Wedd. (syn. *G. verrucosa* V.M. Badillo)	Guángalo	It is used in southern Ecuador to treat skin problems.	Two sesquiterpene lactones, namely, leucodine (**4**) and dehydroleucodine (**5**) (Figure 2), were isolated from an EtOAc extract of the aerial parts [2]. The chemical composition of the EO that was isolated from the leaves via standard distillation in a Clevenger apparatus was reported [30]. α-Zingiberene (45.57 ± 1.66%), α-amorphene (11.12 ± 0.24%), *p*-cymene (15.23 ± 0.10%), and α-phellandrene (11.72 ± 0.15%) were identified using GC/MS as the most abundant components [30].	Dehydroleucodine (**5**) was active against an entire panel of eight different acute leukemia cell lines with an average LD_50_ value of 9.4 μM [6].
*Lasiocephalus ovatus* Schltld.	Arquitecta	To treat venereal diseases and prostate inflammation and used as a diuretic.	Twenty-seven components were identified using GC/MS in the EO that was hydrodistilled from the plant collected in Chimborazo province. The most abundant compounds were camphor (40.48%), 1,2,5,5-tetramethyl-1,3-cyclopenta-diene (11.90%), *p*-mentha-1,5-dien-8-ol (5.23%), and 1,6-dimethyl-hepta-1,3,5-triene (4.69%) [37].	The EO antimicrobial activity, using the broth microdilution technique in 96-well microplates, was tested against *Enterococcus faecalis*, *Pseudomonas aeruginosa*, *Staphylococcus aureus*, *Escherichia coli*, *Klebsiella pneumonia*, and *Candida albicans*. Moderate activity was observed against *S. aureus* (MIC = 200–400 μg/mL) and *E. coli* [37].
*Tagetes filifolia* Lag. *Tagetes terniflora* Kunth *Tagetes minuta* L. *Tagetes multiflora* Kunth *Tagetes zypaquirensis* Bonpl.	Sacha anis (fake anise) or chilchil wandura	*Tagetes* are used as medicinal plants, as well as ornamental plants, food additives, biopesticides, and for dyeing clothes.	The EOs of five species, collected at different locations across Ecuador, were isolated and chemically characterized. The type and the relative abundance of the oil components depended on the geographical origin of the plant. Phenylpropanoids and terpenes were the main oil components, including anethole (66.9%), estragole (31.6%), and anisaldehyde (1.5%) in *T. filifolia*, which were collected in the Cañar, Loja, and Pichincha provinces; *trans*-tagetone (52.7%), 4-ethyl-4-methyl-1-hexene (25.6%), verbenone (3.32%), 1-verbenone (3%), β-ocimene (8.62%), β-linalool (1.19%), and *cis*-tagetone (6.21%) in *T. terniflora*, which were collected in the Cañar, Chimborazo, Pichincha, and Tungurahua provinces; *trans*-tagetone (33.9%), 4-ethyl-4-methyl-1-hexene (13.8%), caryophyllene (3.2%), β-ocimene (16.9%), *trans*-ocimene (3.7%), *cis*-tagetone (9.1%), 1-verbenone (11.69%), and verbenone (16.6%) in *T. minuta*, which were collected in the Pichincha province; *trans*-tagetone (17.89%), *trans*-ocimene (3.73%), 1-verbenone (13.89%), verbenone (24.3%), and 4-ethyl-4-methyl-1-hexene (39.7%) in *T. zypaquirensis*, which were collected in the Bolivar, Cañar, Carchi, Chimborazo, Cotopaxi, Imbabura, and Pichincha provinces; and *trans*-tagetone (30.9%), *trans*-ocimene (25.6%), and valeric acid (43.4%) in *T. multiflora*, which were collected in the Azuay, Cañar, Chimborazo, and Pichincha provinces [38].	
*Balanophoraceae*				
*Corynaea crassa* Hook. f.	Huanarpo male	Aphrodisiac.	Safrole, squalene, sesquiterpenes, steroids, and triterpenes were characteristic components of an EtOAc extract, which was analyzed using GC [39].	Experiments on a carrageenan- induced acute inflammation model showed the anti-inflammatory activity of aqueous and ethanolic extracts. In general, the analysis of samples from Peru and Ecuador gave similar results [39].
Bignoniaceae				
*Mansoa alliacea* (Lam.) A.H. Gentry	Ajo de monte o sacha ajo (fake garlic)	In healing ceremonies and shaman rituals in the Amazon region, in treatments against rheumatism and atherosclerosis, and an analgesic and antipyretic remedy.	The most important compounds that occur in the plant are sulfur derivatives, such as diallyl disulfide, diallyl trisulfide, alliin, allicin, allyl propyl sulfide, divinyl sulfide, diallyl sulfide, and dimethyl sulfide. The presence of a few sterols, iridoids, flavonoids, and alkaloids was also reported [40].	Antibacterial, antifungal, antioxidant, anti-inflammatory, and anti-plasmodial properties were reported [40].
Bryophytes				
Mosses: *Breutelia tormentosa* (SW. ex Brid.) A. Jaeger (Bartramiaceae) *Campylopus richardii* Brid. (Dicranaceae) *Leptodontium viticulosoides* (P. Beauv.) Wijk & Margad. (Pottiaceae) *Macromitrium perreflexum* Steere (an unresolved name) *Rhacocarpus purpurascens* (Brid.) Paris (Rhacocarpaceae) *Thuidium peruvianum* Mitt. (Thuidiaceae) Liverworts: *Frullania brasiliensis* Raddi (an unresolved name) (Jubulaceae), *Leptoscyphus hexagonus* (Nees) Grolle (Geocalycaceae, Lophocoleaceae), *Herbertus juniperoideus* (Sw.) Grolle (Herbertaceae), *Syzygiella anomala* (Lindenb, & Gottsche) Stephani (Jungermanniaceae)	Musgos Hepáticas		Sesquiterpenes and diterpenes were the most abundant components of the EOs that were hydrodistilled from six mosses, although they were different from species to species. Epizonarene (8.7%) and α-selinene (6.7%) were predominant in *Breutelia tormentosa* (SW. ex Brid.) A. Jaeger; *epi*-α-muurulol (15.1%) and α-cadinol (12.5%) predominated in *Campylopus richardii* Brid., selina-3,11-dien-6-α-ol (19.7%) and curcuphenol (10.6%) in *Macromitrium perreflexum* Steere, α-cadinol (36.8%) and α-santalene (8.4%) predominated in *Rhacocarpus purpurascens* (Brid.) Paris, and phytol (21.7%) and valerenol (10.1%) predominated in *Thuidium peruvianum* Mitt., whereas the EO of *Leptodontium viticulosoides* (P. Beauv.) Wijk & Margad. was rich in β-selinene (13.5%) and α-selinene (10.5%) [41]. A similar study was performed on the oils from four liverworts. Sesquiterpenes were the most important components in all species: τ-muurolol (32.14%) and germacrene D (11.98%) predominated in *Frullania brasilensis* Raddi, cabreuva oxide-D (33.7%) and elemol (18.55%) predominated in *Leptoscysphus hexagonus* (Nees) Grolle, and bicyclogermacrene (18.23%) and caryophyllene oxide (18.29%) predominated in *Herbertus juniperoideus* (Sw.) Grolle, whereas silpherfolane-5,7(14)-diene (25.22%) and caryophyllene oxide (8.98%) were abundant in the oil of *Syzygiella anomala* (Lindenb. & Gottsche) Stephani [42].	
Burseraceae				
*Bursera graveolens* (Kunth) Triana & Planch.	Palo santo	To treat arthritis and to cause sweating, and as an analgesic and sedative remedy. The bark is burnt as incense due to the intense woody scent. The EO from trees that grow in the dry forest of Zapotillo “Palo Santo Valley” (Ecuador) was exported by a Brazilian company as a perfume ingredient.	Ninety-nine compounds were identified using GC/MS in the EO hydrodistilled from the stems; limonene (34.9%) and α-terpineol (13.4%) were the main constituents [43]. Sixty-one components were determined using GC/MS in the EO that was hydrodistilled from mature fruits, among which, the most abundant were limonene (49.89%), α-phellandrene (37.64%), and menthofuran (6.08%) [44].	The radical scavenging effect of the stem EO was only moderate in a DPPH test (22.9 ± 2.3 mg/mL) and a value of 179.5 ± 23.4 μM ascorbic acid equivalents was determined using a ferric reducing antioxidant power (FRAP) assay [43]. The EO from mature fruits that were mixed with the EO from *Schinus molle* showed potential acaricidal activity against larvae of the cattle tick *Rhipicephalus* (*Boophilus*) *microplus* [44].
*Dacryodes peruviana* (Loes.) H.J. Lam	Copal, copal comestible, anime, or wigonkawe	The gummy resin is used as an insect repellent.	Twenty-five compounds, including the main one, α-phellandrene (50.32%), were identified using GC/MS in the EO that was hydrodistilled from the fruits [45].	The EO was tested using the microdilution method against the bacteria *Proteus vulgaris*, *Pseudomonas aeruginosa*, *Escherichia coli*, *Klebsiella pneumonia*, *Salmonella typhimurium*, *Staphylococcus aureus*, and *Enterococcus faecalis*, and the fungi *Trichophyton rubrum* and *T. mentagrophytes.* The oil exhibited a modest activity against *S. aureus* (MIC = 625 μg/mL), while the MIC values against the remaining microorganisms were, on average, above 2500 μg/mL. Furthermore, the oil was a potential source of a repellent product against mosquitoes [45].
Cactaceae				
*Hylocereus megalanthus* (K. Schum. ex Vaupel) Ralf Bauer	Yellow pitahaya or dragon fruits	The edible fruits are exported from Ecuador to different countries.	Palmitic (11.52%), stearic (4.29%), oleic (11.09%), vaccenic (3.08%), and linoleic (69.98%) acids were the main fatty acids in the fruits that were collected in the Amazon and extracted in a Soxhlet [46].	
Chloranthaceae				
*Hedyosmum luteynii* Todzia	Sacha guayusa (fake guayusa), borrachero, or tarqui	It is used in Chimborazo province to alleviate respiratory diseases.	Monoterpenes were the main components of the EO that was hydrodistilled from fresh leaves; α-phellandrene (32.72%), α-pinene (13.20%), (*Z*)-β-ocimene (10.99%), silvestrene (6.51%), bicyclogermacrene (5.05%), 1,8-cineol (4.95%), (*E*)-β-ocimene (3.88%), and germacrene D (3.20%) predominated [47].	
*Hedyosmum racemosum* (Ruiz & Pav.) G. Don	Guayusa de monte (thicket guayusa) or jicamilla	Anti-cancer treatment.	Onoseriolide (**6**) (Figure 2) was isolated from the plant [48].	Onoseriolide (**6**) showed interesting antiproliferative effects on a human colorectal-cancer-derived cell line (RKO), causing cell cycle arrest at the G2/M phase and activating both apoptosis, as a cell death mechanism, and autophagy, as a survival mechanism [48].
*Hedyosmum scabrum* (Ruiz & Pav.) Solms	Granizo, tarqui, or guayusa de cerro	Several *Hedyosmum* species are used to treat various diseases and ailments, such as diarrhea, respiratory disorders, and stomachache.	The compositions of the EOs that were hydrodistilled in a Clevenger apparatus from flowers of male and female *H. scabrum* were qualitatively and quantitatively rather different, including the distribution of enantiomers. The main components of the EO from the male specimens were pinocarvone (13.1%), germacrene D-4-ol (12.6%), 1,8-cineole (10.8%), α-pinene (6.4%), and β-pinene (4.8%), while the main constituents of the EO from female flowers were 1,8-cineole (20.5%), linalool (16.5%), α-pinene (15.0%), β-pinene (6.4%), and sabinene (6.3%) [49].	
Clusiaceae				
*Clusia latipes* Planch. & Triana	Duco		Alkaloids, flavonoids, carbohydrates, tannins, and saponins were detected in organic extracts of the plant collected in the Loja province. Isoquercitrin (**7**) (Figure 2) was isolated from the EtOAc extract [50].	A direct relationship between the antioxidant capacity and the α-glucosidase inhibitory activity was demonstrated for the EtOAc extract. This extract exhibited the highest antioxidant activity while producing the strongest enzyme inhibitor with an IC_50_ = 0.90 µg/mL [50].
Ericaceae				
*Bejaria resinosa* Mutis ex L.f.	Pena de cerro	It is used by Saraguros as a first-aid treatment of ailments, such as liver diseases, cancer, swollen wounds, and inflammation of the genital organs.	The triterpenes α-amyrin (**8**), β-amyrin (**9**), taraxerol (**10**), and ursolic acid (**11**), as well as the flavonoid quercetin-3-*O*-rahmmnoside (**12**) (Figure 3), were isolated from organic extracts and identified using NMR techniques [51].	Isolated compounds and an aqueous extract were tested (MTS assay) against MCF-7 (breast carcinoma), PC-3 (prostate carcinoma), RKO (colon cancer), and D-384 (astrocytoma) human tumor cells. The aqueous extract was active against all these lines, except against MCF-7 cells, whereas ursolic acid (**11**) was active against all these lines with IC_50_ ± SEM values (µM) of 10.39 ± 1.46 (D-384), 17.16 ± 8.99 (RKO), 7.43 ± 0.64 (MCF-7), and 12.11 ± 0.52 (PC-3) [51].
Euphorbiaceae				
*Croton ferrugineus* Kunth (syn. *C. elegans* Kunth)	Mosquera	To heal wounds and to treat inflammation, toothache, bronchitis, gout, and rheumatism.	Friedelin (**13**), cycloeucalenol (**14**), (+)-pallidine (**15**), and (+)-*O*-methyl-pallidine (**16**) (Figure 3) were identified using spectroscopic techniques [52].	
*Croton thurifer* Kunth	Mosquera (the name originates from the resin that exudes from the tree)	The exudate latex of the stem bark is used to eliminate warts and to treat wounds, sores and ulcers	(3*R*,20*S*)-3,20-dihydroxydam- mara-24-ene 3-*O*-palmitate (**17**), (3*R*,20*S*)-3,20-dihydroxydam-mara-24-ene 3-*O*-acetate (**18**), *trans*-phytol (**19**), vomifoliol (**20**), β-sitosterol (**21**), *trans*-tiliroside (**22**), and sparsifol (**23**) (Figure 4) were isolated from the plant [53].	A modest hypoglycemic activity was observed for the EtOAc extract using an α-glucosidase inhibitory activity assay (IC_50_ of 1.77 mg/mL); furthermore, *trans*-tiliroside (**22**) (IC_50_ = 114.85 μg/mL) and (3*R*,20*S*)-3,20-dihydroxydam-mara-24-ene 3-*O*-acetate (**18**) (IC_50_ = 292.87 μg/mL) exhibited strong inhibitory activities compared with the positive control acarbose [53].
*Croton wagneri* Müll. Arg.	Mosquera blanca (white mosquera)	It is used by indigenous communities to cure tonsillitis; reduce stomach acidity; and against diabetes, fever, and gastritis.	The main components of the leaf EO were *cis*-chrysanthenol (27.5%), myrcene (19.2%), and *cis*-chrysantenyl acetate (8.6%) [54].	Moderate antioxidant activity was determined for the EO, with a percentage of DPPH radical scavenging activity of 79.5 ± 2.4% and a ferric-reducing antioxidant power (FRAP) of 292.3 ± 24.0 μM as ascorbic acid equivalents [54].
Fabaceae				
*Dalea mutisii* Kunth (syn. *D. coerulea* (L. f.) Schinz & Tell.)	Iso	To treat pneumonia.	The main components of the flower EO, which was rich in monoterpene hydrocarbons, were α-pinene (42.9%), β-pinene (15.1%), β-phellandrene (12.1%), myrcene (6.7%), and (*Z*)-β-ocimene (5.4%). The enantiomeric composition of the oil was also determined [55].	
*Otholobium mexicanum* (L. f.) J.W. (Grimes)	Culen	To treat diarrhea and gastric problems, and as a contraceptive and antibacterial remedy.	Bakuchiol (**24**), hydroxybaku- chiol (**25**), daidzin (**26**), and genistin (**27**) (Figure 5) were isolated from a leaf methanolic extract and identified using NMR spectroscopy [56].	In the α-amylase assay, the methanolic extract exhibited moderate inhibitory activity with an IC_50_ of 470 μg/mL, while inhibition percentages of bakuchiol (**24**), 3-hydroxybakuchiol (**25**), and daidzin (**26**) were less than 25% at the maximum dose that was tested (1 μM). Genistin (**27**) exhibited poor activity with an IC_50_ of 805 μM. In the α-glucosidase assay, the methanolic extract exhibited strong inhibitory activity with an IC_50_ value of 32 μg/mL, while 3-hydroxybakuchiol (**25**) exhibited moderate inhibitory activity, with an IC_50_ of 345 μM. Daidzin (**26**) and genistin (**27**) exhibited lower inhibitory activity, with IC_50_ values of 564 and 913 μM, respectively. Bakuchiol (**24**) exhibited poor inhibitory activity, with an inhibition percentage less than 10% at the maximum dose tested (1 mM) [56].
Elaeocarpaceae				
Vallea stipularis *L. f.*	Several popular names, depending on the place of growth: chuillur, cugur, rosa, achiotico, achacapuli, and campano are the most common ones	A remedy against gastritis, inflammation, scurvy, and rheumatism.	The flavonoid glycoside *trans*-tiliroside (**22**) (Figure 4) was isolated from the leaves [1].	*trans*-Tiliroside (**22**) showed an interesting selective BuChE inhibitory activity (IC_50_ = 52.9 μM) [1].
Hypericaceae				
*Hypericum decandrum* Turcz. *Hypericum laricifolium* Juss. *Hypericum quitense* R. Keller	Bura chica (Azuay province), matikillkana, bura de llano, san Juan, romerillo	*H. laricifolium* is mainly used to cure antibacterial infections.		Research was performed with the aims to (i) develop a predictive model for the antibacterial potential of the genus *Hypericum* using HPLC fingerprints of *H. laricifolium*, *H. quitense*, and *H. decandrum* that were collected in the Andean regions of Azuay, Loja, and Cañar (Ecuador); and (ii) evaluate the influence of natural variables, such as the altitude and soil composition on the pharmacological effect of lipophilic extracts of *H. laricifolium*. They exhibited high antibacterial activity, with the pH, soil components, and organic matter as the main factors that influenced the activity; furthermore, no relationship was found between altitude and the antibacterial effects. The prediction model that was obtained did not have predictive ability for different *Hypericum* species, which could be explained by the differences in the chemical compositions of the three species [57].
Lamiaceae				
*Clinopodium brownei* (Sw.) Kuntze	Poleo chico or poleo warmi chico in Kichwa	Poleo warmi is used by the Saraguros as a digestive and to relieve the discomfort of menstrual colic. It is also considered an effective expectorant agent, and a remedy to cure colds, flu, cough, bronchitis and asthma	Thirty-one components, accounting for 96.15% of the oil that was hydrodistilled from the plant, were identified using GC/MS. The main components were pulegone (48.44%), menthone (34.55%), and β-acorenol (3.41%). Oxygenated monoterpenes (86.06%), followed by oxygenated sesquiterpenes (5.36%), constituted the most abundant fractions. The enantiomeric compositions of β-pinene, sabinene, 3-octanol, menthone, pulegone, and methyl acetate were determined using enantioselective GC/MS [58]. (−)-Menthone showed the highest enantiomeric excess (ee = 83.4%) [58].	In in vitro tests, the EO showed high selective inhibitory activity against BuChE, with an IC_50_ = 13.4 ± 1.8 mg/mL. In contrast, it was weakly active against AChE with an IC_50_ > 250 µg/mL [58].
*Lepechinia heteromorpha* (Briq.) Epling	Shalshon or zhalshon in Kichwa		Twenty-five constituents were identified in the leaf EO using GC/FID and GC/MS. (−)-Ledol (28) (21.2%) and (−)-caryophyllene oxide (**29**) (1.0%) (Figure 6) were also isolated and their structures were confirmed using NMR spectroscopy. Other main constituents of the EO were viridiflorene (27.3%), (*E*,*E*)-farnesene (1.4%), spirolepechinene and (*E*)-caryophyllene (7.1% each), allo-aromadendrene (6.1%), camphor (1.7%), limonene (1.3%), and β-phellandrene (4.6%). The enantiomeric excesses and distribution of α-pinene, limonene, β-phellandrene, and camphor were also determined [59].	
*Lepechinia mutica* (Benth.) Epling	Shalshon in Kichwa or casa casa in Spanish	Espanto (startle)	Seventy-nine components, accounting for 97.3% of the sample, were identified and quantified using GC/FID and GC/MS in the EO from the leaves. Sesquiterpene hydrocarbons (38.50%) and monoterpene hydrocarbons (30.59%) were the most abundant volatiles, whereas oxygenated sesquiterpenes (16.20%) and oxygenated monoterpenes (2.10%) were the minor components. Moreover, the most important odorants, from the sensory point of view, were identified using aroma extract dilution analysis (AEDA) GC/O. α-Pinene, β-phellandrene, and dauca-5,8-diene exhibited characteristic woody, herbaceous, and earthy odors, respectively. Twelve enantiomeric pairs and two enantiomerically pure chiral monoterpenoids were revealed using enantioselective GC analysis of the oil. The enantiomeric excesses varied from a few percent units to virtually 100% [60]. Ursolic acid (**8**), oleanolic acid (**9**) (Figure 3), carnosol (**30**), viridiflorol (**31**), chrysothol (**32**), and 5-hydroxy-4′,7-dimethoxy flavone (**33**) (Figure 6) were identified in the non-volatile fraction from the leaves of the plant. Their structures were determined using X-ray diffraction and NMR and MS techniques [60]. The chemical compositions of the EOs from the flowers and the leaves were similar, except for several minor components. The main constituents (>4%) of the flower EO were δ-3-carene (24.23%), eudesm-7(11)-en-4-ol (13.02%), thujopsan-2-α-ol (11.90%), β-pinene (7.96%), valerianol (5.19%), and co-eluted limonene and β-phellandrene (4.47%). Enantiomeric pairs of α-thujene, β-pinene, sabinene, α-phellandrene, limonene, and β-phellandrene were identified using enantioselective analysis on a β-cyclodextrin column [61].	The leaf EO exhibited moderate in vitro activity against five fungal strains, with it being especially effective against *Microsporum canis*, which is a severe zoophilic dermatophyte that is a causal agent of pet and human infections [60]. The diterpene carnosol (**30**) (Figure 6) exhibited high activity against the “blast disease” that is caused by the fungus *Pyricularia oryzae* [61]. The minimum inhibitory concentration (MIC) and the minimum fungicidal concentration (MFC) values of carnosol (**30**) against this fungus were very close to those of the well-known pesticide flutriafol [61]. Moreover, carnosol (**30**) showed a promising selective inhibitory activity of the enzyme BuChE (5.15 M, in comparison with 8.568 ± 0.570 M of the positive control donepezil) [1].
*Lepechinia paniculata* (Kunth) Epling	Yayllon or llanllum in Kichwa	The buds are tied at the forehead for treating the “mal de aire”, a sort of evil eye, and against headache, while flower infusions are used to treat nervous diseases.	Column chromatographic separation of the leaf EtOAc extract afforded ledol (**28**), (−)-caryophyllene oxide (**29**), (−)-carnosol (**30**), and guaiol (**34**) (Figure 6) [59]. In another work, 40 and 29 compounds were identified in the EOs that were steam distilled from the leaves and the flowers, respectively. The main components of the oils were 1,8-cineole, β-pinene, δ-3-carene, α-pinene, (*E*)-caryophyllene, guaiol (**34**), and β-phellandrene [62].	The flower EO showed interesting inhibitory activity against the enzymes AChE (IC_50_ = 28.2 ± 1.8 2 µg/mL) and BuChE (IC_50_ = 28.8 ± 1.5 µg/mL). By contrast, the leaf EO showed moderate inhibitory activity against the two enzymes, with IC_50_ values of 38.2 ± 2.9 µg/mL (AChE) and 47.4 ± 2.3 µg/mL (BuChE) [62].
*Lepechinia radula* (Benth.) Epling	Shalshon or zhalshon in Kichwa	The leaves are used to treat “mal de aire” and aches in muscles and bones	Column chromatographic separation of the leaf EtOAc extract afforded 5-hydroxy-4′,7-dimethoxy flavone (**33**), spathulenol (**35**), and angustanoic acid E (**36**) (Figure 6) [59]. Thirty-four compounds were identified in the EO, accounting for 93.4% of the oil. The main constituents were δ-3-carene (19.9%), β-pinene (17.0%), (*E*)-β-caryophyllene (9.7%), and (*E*,*E*)-α-farnesene (9.4%) [63].	The EO exhibited strong antifungal activity against *Trichophyton rubrum* and *T. mentagrophytes* [63].
*Melissa officinalis* L.	Toronjil	Relaxant, insomnia	The ethanol extract, which was obtained via maceration of the leaves, was a rich source of essential fatty acids and derivatives, benzenoids, phytosterols, and pentacyclic triterpenes [64].	On the basis of the literature, most constituents identified using GC/MS showed interesting biological activities, including antimicrobial and antitumor properties [64].
*Ocimum campechianum* Mill.	Albahaca (basil) or albahaca blanca (white basil)	This plant is used by indigenous population both for culinary and medicinal purposes	The volatile fractions from the methanol and the 70% aqueous ethanol extracts of the aerial parts of the plant were chemically characterized using GC/MS and HPLC-DAD-MS techniques [65].	The EO, the raw extracts, and the main constituents, namely, eugenol and rosmarinic acid, showed significant IC_50_ values in the DPPH and ABTS assays. The EO and eugenol also showed remarkable activity against *Pseudomonas syringae pv. syringae* and moderate effects against *Candida* spp. clinical isolates, with a possible antimicrobial synergy in association with fluconazole. The extracts and isolated compounds were weakly cytotoxic against a HaCaT cell line (keratinocytes) and non-mutagenic against *Salmonella typhimurium* TA98 and TA100 strains, indicating safety. The EO was weakly active against human adenocarcinoma alveolar basal epithelial cells (A549 cell line). This evidence suggests a potential use of the crude drug, extracts, and the EO as antioxidant agents in cosmetic formulations and food supplements. In addition, the EO may also have potential applications in plant protection and anti-*Candida* formulations [65].
*Salvia leucantha* Cav.	Salvia or salvia morada (Mexican bush sage or velvet sage)	The plant is used in traditional medicine as a remedy to relieve cough, chest, lung, and stomach pains, as well as a garden plant	Six main compounds, namely, 6,9-guaiadiene (19.14%), (*E*)-caryophyllene (16.80%), germacrene D (10.22%), (*E*)-β-farnesene (10.00%), bicyclogermacrene (7.52%), and bornyl acetate (14.74%), were identified using GC/MS and GC/FID in the EO that was steam distilled from the aerial parts. Four pairs of enantiomers were determined using enantioselective GC/MS on the EO. (−)-Germacrene D and (+)-α-pinene showed the highest ee [66].	In an in vitro assay, the EO exhibited interesting inhibitory activity of the enzyme BuChE, with an IC_50_ *=* 32.60 µg/mL, which was the best value that was determined for an oil from a *Salvia* species. In contrast, the oil was weakly active against AChE, with an IC_50_ > 250 µg/mL [66].
*Salvia pichinchensis* Benth.	Matico de cerro or quinde- sungana-mangapaque	The leaves are used for curing kidney and liver disorders, headache and to treat the infection of external wounds	*cis*-Cadina-1(6), 4-diene (17.11%), γ-curcumene (13.75%), (*E*)-caryophyllene (12.58%), (*E*, *E*)-α-farnesene (10.00%), α-gurjunene (9.46%), and allo-aromadendrene (6.96%) were identified as the main components of the EO distilled from the aerial parts [67].	The EO showed interesting selective inhibitory activity against the enzyme BuChE (IC_50_ *=* 50.70 μg/mL) and only low inhibitory activity against AChE (IC_50_ *=* 117.60 μg/mL) [67].
*Salvia sagittata* Ruiz & Pav.	Salvia, salvia hoja de flecha	To treat inflammation and different intestinal ailments, fever, influenza, gastritis, cuts, and bumps		The effects of an ethanolic extract of *S. sagittata* (SSEE) on primary cultures of porcine aortic endothelial cells (pAECs) were investigated. The cells were cultured in the presence of different concentrations (1–200 μg/mL) of SSEE for 24 h, and the cytotoxicity was evaluated using a 3-(4,5-dimethyl-2- thiazolyl)-2,5-diphenyl-2-H-tetrazolium bromide (MTT) assay. SSEE did not adversely affect the cellular viability at any tested concentration. No significant change was observed in the cell cycle. The anti-inflammatory effects of SSEE on pAECs were analyzed using a lipopolysaccharide (LPS) as the inflammatory stimulus. Different markers that were involved in the inflammatory process, such as cytokines and protective compounds, were evaluated using real-time quantitative PCR and Western blots. SSEE showed the ability to restore pAEC physiological conditions by reducing interleukin-6 and increasing heme oxygenase-1 protein levels. The phytochemical composition of SSEE was also evaluated via HPLC/DAD and spectrophotometric assays. The presence of different phenolic acids and flavonoids was revealed, including rosmarinic acid as the most abundant component. SSEE possessed an interesting antioxidant activity, as assessed using the oxygen radical absorbance capacity (ORAC) and DPPH assays. In conclusion, SSEE was suggested to have in vitro anti-inflammatory effects. This finding represents the initial step toward possible scientific support for the traditional therapeutic use of the plant [68].
Lauraceae				
*Ocotea quixos* (Lam.) Kosterm.	Ishpingo	In the preparation of aromatic beverages to which different health benefits are attributed.	A total of 112 volatiles were identified in the leaf EO, among which, 1,8-cineole (21.4%) and *p*-cymene (12.6%) predominated [69]. Another study demonstrated the variability of the chemical composition of the EO isolated from Amazonian *O. quixos*. Forty-seven compounds were identified using GC/MS and GC/FID, which represented between 97.17 and 99.89% of the oil composition. The constituents were grouped in aliphatic sesquiterpene hydrocarbons (33.03–55.89%), oxygenated monoterpenes (1.97–39.66%), and other compounds (8.94–47.83%). The main constituents were (*E*)-cinnamyl acetate (5.96–41.65%), (*E*)-methyl cinnamate (0.38–37.91%), and (*E*)-caryophyllene (8.77–37.02%). Statistical analysis suggested the existence of two EO chemotypes and a direct correlation between the environmental conditions and the chemical composition of the oils [70].	The EO showed moderate antioxidant activity in the DPPH and the FRAP assays. Concerning the oil antimicrobial activity, the MICs were 0.5 μL/L for *Staphylococcus aureus*, 0.05 μL/L for *Bacillus subtilis*, 5 μL/L for *Escherichia coli*, 0.05 μL/L for *Salmonella enteritidis*, 0.5 μL/L for *Aspergillus niger*, and 0.5 μL/L for *Pennicillium citrinum* [69]. In another investigation, the leaf EO was used to predict the termiticidal and repellent effects on termites *Nasutitermes corniger* using a one-factor response surface methodology design. The variable that was analyzed was the concentration of the EO in EtOH at an interval of 0.05–0.3% for the anti-termite activity and between 0.01 and 0.12% for the repellent action. A 100% mortality rate was found at oil concentrations > 0.12%, while the effect was 22.2% at the minimum concentration analyzed. Moreover, 100 and 48.9% of the termites were repelled by the oil at concentrations of 0.12 and 0.01%, respectively. Forty-two compounds, 39 of which were identified, were detected in the leaf EO, which was analyzed using GC/MS. The main compounds were (*E*)-cinnamyl acetate (36.44%), (*E*)-cinnamaldehyde (27.03%), (*E*)-β-caryophyllene (5.21%), and (*E*)-methyl isoeugenol (4.18%) [71].
Lecythudaceae				
*Grias neuberthii* J.F. Macbr.	Pitón in Pastaza province	Presumed antiproliferative activity against tumor cells.		The cytotoxic effects of leaf, seed, fruit, stem, and bark extracts on colon carcinoma cells RKO (normal p53) and SW613-B3 (mutated p53) were investigated. The stem bark methanolic extract exhibited the highest cytotoxic potential. Moreover, the cytotoxic effect was similar on both cell lines, indicating that it was independent of the status of p53. However, RKO cells were more sensitive than SW613-B3 cells. No evidence for apoptotic markers was recorded; nevertheless, both cell lines showed signs of autophagy after the treatment, including increased Beclin-1 and LC3-II and decreased p62. Lupeol (**37**), 3′-*O*-methyl ellagic acid 4-*O*-β-D-rhamnopyranoside (**38**), and 19-α-hydroxyasiatic acid 27-*O*-β-D-glucopyranoside (**39**) (Figure 7) were identified as the compounds that were likely responsible for the activity [72].
Loranthaceae				
*Gaiadendron punctatum* (Ruiz & Pav.) G. Don.	Violeta de campo (field violet)	In the traditional medicine of the Saraguro community.	Five quercetin glycosides and one kaempferol glycoside were isolated from hydroalcoholic extracts of leaves and flowers. In addition to nicotiflorin (**40**) from flowers, rutin (**41**) from flowers and leaves, and artabotryside A (**42**) from leaves, three novel quercetin glycosides were isolated: hecpatrin (**43**) and gaiadendrin (**44**) from the leaves, and puchikrin (**45**) from the flowers (Figure 7) [73].	The leaf hydroalcoholic extract exhibited antimicrobial activity against *Micrococcus luteus*, *Staphylococcus aureus*, and *Enterococcus faecalis*, whereas the flower hydroalcoholic extract was only active against *Micrococcus luteus*. In striking contrast, flavonoid glycosides were weakly active against bacteria. Moreover, the hydroalcoholic extracts and the flavonoids inhibited the activity of α-glucosidase in a dose-dependent manner. Nicotiflorin (**40**) rutin (**41**) and gaiadendrin (**44**) were competitive α-glucosidase inhibitors, while hecpatrin (**43**) was a non-competitive inhibitor [73].
Lycopodiaceae				
*Huperzia brevifolia* (Grev. & Hook.) Holub *Huperzia columnaris* B. Øllg. *Huperzia compacta* (Hook.) Trevis. *Huperzia crassa* (Humb. & Bonpl. Ex Willd.) Rothm. *Huperzia espinosana* B. Øllg. *Huperzia tetragona* (Hook. & Grev.) Trevis. *Huperzia weberbaueri* (Hieron. & Herter ex Nessel) Holub	Waminga verde (green waminga), waminga oso (bear waminga), waminga roja (red waminga), waminga amarilla (yellow waminga), waminga oso warmi (female bear waminga), trencilla roja (red trencilla), or waminga suca (light grey waminga)	Considered sacred by the Saraguros [4], these plants are widely used as intestinal purgative remedies and in ritual ceremonies. Mixed with other plants, some species also induce a state of trance or hallucinations in participants in magical–religious rituals [4].	GC/MS analysis of the volatile alkaloidal fractions led to the identification of a few lycodine-type and lycopodine-type alkaloids (**46**–**50**) (Figure 8) in *H. compacta*, *H. columnaris*, and *H. tetragona*. The flavones selgin (**51**) and tricin (**52**) (Figure 8) were isolated from *H. brevifolia* and *H. espinosana*, and tricin (**52**) was also detected in the other five species. The rare serratene triterpenes serratenediol (**53**), serratenediol-3-*O*-acetate (**54**), 21-episerratene-diol (**55**), and 21-episerratenediol 3-*O*-acetate (**56**) (Figure 8) were isolated from *H. crassa*. In addition, the presence of unprecedented high-molecular-weight alkaloids was determined. An analytical UHPLC-UV-MS method for the quantification of tricin (**52**) in the extracts of *Huperzia* plants was also described [74].	The significant AChE and monoamine oxidase A (MAO-A) inhibitory activity of the alkaloidal fractions from *H. brevifolia*, *H. compacta*, *H. espinosana*, and *H. tetragona* may support the use of these plants to prepare brews that induce psychoactive effects in participants in magical–religious ceremonies [4]. The unusually high amount of tricin (**52**) in *H. brevifolia* and *H. compacta* is remarkable, where this flavone is considered a potent selective inhibitor of different cancer cell lines and a potential colorectal cancer chemopreventive agent [74].
Malvaceae				
*Malva pseudolavatera* Webb & Berthel. *Malva sylvestris* L.	Malva (mallow), malva loca, malva alta, malva lisa, or malva mayor	Mucolytic	The hexane and the 80% hydroalcoholic extracts of the leaves were analyzed using GC/MS. The phytochemical contents of the two species were similar and included fatty acids, diterpenes and triterpenes, phytosterols, and abundant amino acids [75].	The leaf aqueous extracts of the two species showed an important mucolytic effect, confirming the traditional use. Thus, the two *Malva* extracts are potential sources of vegetable material for research and development of phytotherapeutic products with mucolytic and gastroprotective activities [75].
Myrtaceae				
*Eucalyptus globulus* Labill.	Eucalipto (eucalyptus)	The leaves are used to treat colds, flu, and coughs	Two EOs were isolated, with yields of 0.17 and 0.15% (*v*/*m*), respectively, via hydrodistillation of leaves collected in the canton Cañar, in the regions Moyancón and Chorocópte at altitudes of 1347 and 3191 m above sea level, respectively. Ninety-nine compounds were identified and quantified in the oils using GC/MS and GC/FID. The compositions of the two EOs were not significantly different, with 1,8-cineole and α-pinene being the main components [76].	The EO from the Moyancón region showed moderate antibacterial activity against *Staphylococcus aureus*, *Streptococcus pyogenes*, and *Escherichia coli* [76].
*Myrcia mollis* (Kunth) DC.	Geberber	The fruits are edible	A total of 22 compounds were identified in the EO. The main components (>5.0%) were α-pinene (27.7–29.2%), β-pinene (30.0–31.3%), myrcene (5.0–5.2%), 1,8-cineole (8.5–8.7%), and linalool (7.7–8.2%). The enantiomeric excess of five chiral constituents was determined. (*S*)-α-pinene and (+)-germacrene D were enantiomerically pure. β-Pinene, 1,8-cineole, γ-terpinene, terpinolene, linalool, and (*E*)-β-caryophyllene were mainly responsible for the aroma of the oil [77].	
*Myrcia splendens* (Sw.) DC. (syn. *M. fallax* (Rich.) DC.)	Capulincillo		The main components of the EO were *trans*-nerolidol (67.81%) and α-bisabolol (17.51%) [78].	The EO cytotoxic activity was tested (MTT) against MCF-7 (breast), A549 (lung) human tumor cell lines, and a HaCaT (human keratinocytes) non-tumor cell line. A promising selective and efficient activity was observed against the MCF-7 cell line (IC_50_ = 5.59 ± 0.13 g/mL at 48 h), which was mainly due to the high content of α-bisabolol in the oil. Weak antibacterial effects against Gram-positive and Gram-negative bacteria were observed using a high-performance thin-layer chromatography (HPTLC) bioautographic assay and the microdilution method; *trans*-nerolidol and β-cedren-9-one were mainly responsible for the activity. Equally negligible was the radical scavenging activity, which was measured using the HPTLC bioautographic and spectrophotometric DPPH tests. In contrast, the minimum inhibitory concentration (MIC) values against some phytopathogen strains were remarkable [78].
*Myrcianthes fragrans* (Sw.) McVaugh	Arrayán aromático	A natural aromatic additive that is used in the preparation of the traditional fruit juice *colada morada*, which is typically drunk on the Day of the Dead or All Souls´ Day.	EOs were hydrodistilled from aerial parts that were collected at Cerro Villonaco (Loja-Ecuador) at different phenological growth stages, i.e., during foliation (Fo), flowering (Fl), and fruiting (Fr) periods. A total of 37, 46, and 38 compounds, accounting, respectively, for 96.5, 96.2, and 95.6% of the Fo, Fl, and Fr oils, were identified using GC/MS and GC/FID. Oxygenated monoterpenes were the main components with percentages of 63.1 (Fo), 49.4 (Fl), and 61.9% (Fr), respectively. The main constituents of the oils were the monoterpene aldehydes geranial and neral, the content of which depended on the phenological development stage of the plant, spanning from 31.1 and 23.6% (Fo), to 23.6 and 17.8% (Fl), and 29.7 and 24.3% (Fr), respectively. The high concentration of the mixture of the two aldehydes (citral) makes the aroma of *colada morada* prepared in southern Ecuador quite different from the aromas of the same beverage made in other regions of the country [7].	The pleasant aromatic properties and the good in vitro antimicrobial activity of *arrayán* suggest a plausible scientific explanation for the use of the plant to aromatize a traditional beverage and as a natural anti-infective and anti-yeast agent. The EO may become a novel rich source of the important industrial chemical citral [7].
*Myrcianthes hallii* (O. Berg) McVaugh	Arrayán or arrayán de Quito	As an antiseptic	Thirty-eight compounds were identified in the hydro-methanol extract using ultra-high-performance liquid chromatography (UHPLC) that was hyphenated to heated electrospray ionization MS and UV detectors. They included polyphenols and organic acids [79].	The hydro-methanol extract showed modest antibacterial activity against methicillin-resistant and methicillin-susceptible *Staphylococcus aureus* and multidrug-resistant and susceptible *Pseudomonas aeruginosa*, *Enterococcus* spp., and *Streptococcus pyogenes* strains, with the exception of *E. coli*, which was found to be less sensitive. Interestingly, no relevant differences were observed between methicillin-susceptible and methicillin-resistant strains. Considering the long-standing use of the plant in folk medicine, suggesting the relative safety, the mixture of plant polyphenols has potential interesting bio-medical applications as a natural antiseptic agent through the incorporation in new anti-infective biomaterials and nanomaterials [79].
*Myrcianthes myrsinoides* (Kunth) grifo	Arrayán	Toothache	A total of 58 compounds were identified in the EO using GC/MS and GC/FID. The main components (>5.0%) were limonene (5.2–5.3%), 1,8-cineole (10.4–11.6%), (*Z*)-caryophyllene (16.6–16.8%), *trans*-calamenene (14.6–15.9%), and spathulenol (6.2–6.5%). α-Pinene, β-pinene, (+)-limonene, γ-terpinene, terpinolene, linalool, β-elemene, and spathulenol were identified using the gas chromatography-olfactometry (GC/O) technique as the compounds responsible for the aromatic profile. The enantiomeric excess of eight chiral constituents was determined using enantioselective GC, whereas (+)-limonene and (+)-germacrene D were enantiomerically pure [77].	The EO showed interesting cholinesterase inhibitory activity (IC_50_ = 78.6 μg/mL against AChE and IC_50_ = 18.4 μg/mL against BuChE) [77].
*Myrteola phylicoides* (Benth.) Landrum	Romero blanco de cerro or romero de cerro	For the treatment of fever, cold, measles and “mal aire” (a supernatural disease caused by strong winds)	Thirty-seven compounds, representing 90.30% of the total content, were identified in the EO that was hydro-distilled from the plant. Monoterpene hydrocarbons (53.06%) and sesquiterpene hydrocarbons (35.24%) were the major groups. The main components were α-pinene (30.94%), (*E*)-caryophyllene (21.93%), β-pinene (14.45%), and α-humulene (9.56%) [80].	The EO showed weak in vitro inhibitory activity against AChE (IC_50_ = 60.8 μg/mL) and very low BuChE inhibitory activity (IC_50_ > 250 μg/mL) [80].
Orchidaceae				
*Maxillaria densa* Lidl.	Orquídea de mandíbulas	Antispasmodic and antidiarrheal remedy, and to treat stomach pains; also used as an ornamental plant.	Phenanthrene derivatives were found in methanol and chloroform extracts [81].	Spasmolytic, antinociceptive, anti-inflammatory, and vasorelaxant activities were reported [81].
Oxalidaceae				
*Oxalis tuberosa* Molina	Oca	Remarkable anti-inflammatory properties; used to treat fever, earache, and dermatitis.		The antioxidant activity (µM TE/mg) of a tuber extract was 75.1 ± 6.2 in an ABTS test and 94.7 ± 18.1 in a DPPH assay [20].
Onagraceae				
*Fuchsia hybrida* hort. Ex Siebert & Voss	Pena pena	Relaxant and disinfectant remedy.		The alkaloidal extract exhibited an IC_50_ value of 90 µg extract/mL and 160 µg extract/mL in ABTS and DPPH assays, respectively [20].
*Ludwigia peruviana* (L.) H. Hara	Mejorana de campo	Diuretic and part of a treatment for kidney problems.		The methanolic extract showed an IC_50_ = 80 µg extract/mL and an IC_50_ = 90 µg extract/mL in ABTS and DPPH assays, respectively [20].
Passifloraceae				
*Passiflora ligularis* Juss.	Granadilla	External and internal inflammation, hepatic pain, high cholesterol, scurvy, and high blood pressure	The main components of the EOs from shells, juice, and seeds were squalene (34.92%), pentadecanal (15.28%), and ionol (19.16%), respectively. These phytochemical findings might provide added value to the fruit waste and improve the production chain [82].	Extracts of species belonging to this family showed antifungal, antioxidant, and antibacterial activities.
Piperaceae				
*Peperomia inaequalifolia* Ruiz & Pav.	Congona, conguna, tigresillo	Analgesic, antiparasitic, and sedative effects. When mixed with other plants, it is used to make a traditional drink called *horchata*.	Safrole (32.10%), 11-αH-himachal-4-en-1-β-ol (25.29%), myristicin (13.29%), elemicin (10.07%), and viridiflorol (5.24%) were found in the EO isolated from fresh leaves [83].	The EO showed an interesting antiradical activity in a DPPH test (IC_50_ = 2.220 ± 0.06 mg/mL), antioxidant activity in a photochemiluminescence (PCL) assay (82.8 µM TE g^−1^), antibacterial effects against the Gram-positive bacteria *Staphylococcus aureus* and *Streptococcus mutans* (MIC = 0.10 mg/mL for each strain), and antifungal activity against the yeasts *Candida tropicalis* and *C. albicans* (MIC = 0.10 mg/mL for each microorganism) [83].
*Piper barbatum* Kunth	Cordoncillo allupa	Used by Quichua communities as an antibacterial agent.	The main constituents that were identified in the EO hydrodistilled from the leaves were α-phellandrene (43.16%), *trans*-sesquisabinene hydrate (8.23%), elemol (7.21%), and limonene (7.04%) [84].	The EO showed moderate antimicrobial activity against *Staphylococcus aureus* (MIC = 264 µg/mL), *Streptococcus mutans* (MIC = 132 µg/mL), *Candida albicans* (MIC = 132 µg/mL), and *C. tropicalis* (MIC = 264 µg/mL). In addition to elemol (**57**) and *trans*-sesquisabinene hydrate (**58**) (Figure 9), the major contribution to the antimicrobial activity was due to two minor sesquiterpene alcohols that were present in the EO, namely, β-eudesmol (**59**) (3.49%) and 10-epi-γ-eudesmol (**60**) (1.07%) (Figure 9) [84].
*Piper carpunya* Ruiz & Pav. (syn. *Piper lenticellosum* C. DC.)	Guaviduca	Used in the preparation of a traditional drink called *guaviduca*.	Twenty-eight components were identified in the EO that was hydrodistilled from fresh leaves and spikes; the main ones were piperitone (33.97%), 1,8-cineole (11.92%), limonene (11.07%), safrole (8.18%), and α-pinene (4.49%). Hydrodistillation of leaves and liquid–liquid extraction of *guaviduca* afforded two EOs. The main constituents were 1,8-cineole in the leaf oil, with a percentage ranging from 25.20% in a winter sample to 17.45% in a summer one, and safrole in the traditional beverage, with a percentage ranging from 2.43% in a winter sample to 13.18% in a summer one. In addition, the enantiomeric pairs of sabinene, phellandrene, linalool, and α-terpineol were detected using enantioselective analysis [85].	The EO antibacterial activity was evaluated against *Staphylococcus aureus* (MIC = 100 µL/mL), *Escherichia coli* (MIC = 200 µL/mL), and *Klebsiella pneumoniae* (MIC = 300 µL/mL) [85]. The leaf EO exhibited high AChE inhibitory activity (IC_50_ = 36.42 ± 1.15 µg/mL) [11].
*Piper coruscans* Kunth	Matico	The leaves are used as a purgative.	Fifty-two constituents were identified in the EO, of which, the main ones were (*E*)-β-caryophyllene (**61**) (24.1–25.0%) (Figure 9), α-humulene (11.6–12.0%), caryophyllene oxide (**29**) (9.3–10.9%), linalool (4.5–5.2%), humulene epoxide II (3.6–4.1%), (*E*)-nerolidol (**62**) (3.7–4.0%) (Figure 9), α-copaene (3.7–3.9%), α-muurolol (3.4–3.7%), α-selinene (3.4–3.5%), and β-selinene (3.1–3.3%). Five enantiomer pairs were identified in the EO; the main stereoisomers were (1*S*,5*S*)-(−)-α-pinene (60.0–69.6%), (1*S*,5*S*)-(−)-β-pinene (5.2–7.2%), (*R*)-(−)-α-phellandrene (72.5–78.2%), (*R*)-(+)-limonene (28.6%), and (*R*)-(−)-linalool (1.8–3.1%). Chemical analysis of the hydrolate showed the presence of linalool with a concentration of 12.3–15.7 mg/100 mL [86].	
*Piper ecuadorense* Sodiro	Matico de monte	Disinfectant, healing of wounds	The main components of the EO were bicyclogermacrene (12.98%), 3-thujopsanone (11.59%), α-phellandrene (6.89%), (*E*)-nerolidol (**62**) (6.88%), δ-elemene (6.83%), and shyobunol (5.79%) [87]. The flavanone pinocembrin (**63**) (Figure 9) was isolated from a leaf aqueous extract; the pinocembrin content in a leaf ethanolic extract was estimated to be 6.64 ± 0.17 µg/mL using an HPLC-DAD method [88].	The EO showed significant antimicrobial activity against *Staphyloccocus aureus* (MIC = 250 µg/mL) and remarkable effects against *Trichophyton mentagrophytes* and *T. rumbus* (MIC = 62.5 µg/mL). The antioxidant activity of the oil had an IC_50_ = 1.81 ± 0.09 mg/mL (ABTS) [87]. Pinocembrin (**63**) exhibited antitumor, antimicrobial, anti-inflammatory, and antioxidant properties.
*Piper lanceifolium* Kunth	Matico, hoja de platanillo	A leaf aqueous infusion is taken as a bath to treat skin infections and a leaf decoction mixed with a little alcohol is drunk to treat headache and body pain.	The flavanone sakuranetin (**64**) and two benzoic acid derivatives, namely, lanceaefolic acid methyl ester (**65**) and cyclolanceaefolic acid methyl ester (**66**) (Figure 9), were isolated from the EtOAc extract of dried leaves. Thirty-five compounds were identified in the volatile fraction of the plant, whose main constituents were safrole (48.3%), apiole (13.6%), γ-terpinene (4.1%), (*E*)-β-ocimene (3.9%), and *epi*-α-cadinol (2.9%) [89].	The EO showed moderate antibacterial activity against *Klebsiella pneumoniae* (MIC = 500 µg/mL) [89].
*Piper pseudochurumayu* Ruiz & Pav.	Ámbar ámbar or matico	Used to provide analgesic, diuretic, digestive, dermatological, anthelmintic, antirheumatic and antidiarrheal effects and treat respiratory infections.		The extract exhibited an antioxidant capacity of 790.1 ± 1.3 and 949.3 ± 11.8 µM TE/mg extract in ABTS and DPPH assays, respectively [20].
*Piper pubinervulum* C. DC.	Matico	Used by the indigenous communities living in the Morona Santiago province as an antirheumatic and analgesic remedy, and as an antidote for snake bites.	Forty-four constituents were identified in the EO, whose main components were β-caryophyllene (**61**) (13.18%), γ-asarone (**67**) (8.81%), nerolidol (**62**) (8.54%), and isoeugenol methyl ether (**68**) (7.56%) (Figure 9) [90].	The EO exhibited high antifungal activity against *Candida tropicalis* (MIC = 0.77 mg/mL) and *C. albicans* (MIC = 0.33 mg/mL) [90].
*Piper subscutatum* (Miq.) C. DC.	Matico de monte	Treat wounds	The main components that were identified in the volatile fraction were (*E*)-β-caryophyllene (**61**) (25.2–25.3%), β-chamigrene (7.8–10.3%), (*E*)-nerolidol (**62**) (7.7–8.1%), β-selinene (7.2–7.7%), δ-cadinene (2.7–3.9%), bicyclogermacrene (2.4–3.7%), and β-pinene (2.6–3.4%). Four pairs of enantiomers were determined in the EO using enantioselective GC/MS analysis. The main enantiomers were (1*R*,5*R*)-(+)-α-pinene (ee 28.8%), (1*S*,5*S*)-(–)-β-pinene (ee 77.8%), (*S*)-(–)-limonene (ee 18.4%), and (1*R*,2*S*,6*S*,7*S*,8*S*)-(–)-α-copaene (ee 6.0%). The main compounds that were present in the hydrolate were 6-methyl-5-hepten-2-one (63.7–64.4%) and linalool (6.5–6.0%). Five lignans were isolated from the nonvolatile fraction and identified as (–)-beilshminol B (**69**), (–)-grandisin (**70**), (–)-3′,4′-methylenedioxy-3,4,5-trimethoxy-7,7′-epo-xylignan (**71**), (–)-3′,4′-methylenedioxy-3,4,5,5′-tetramethoxy-7,7′-epoxylignan (**72**), and (–)-3,4,3′,4′-dimethylene-dioxy-5,5′-dimethoxy-7,7′-epoxylignan (**73**) (Figure 10) [91].	
*Sarcorhachis sydowii* Trel.	Called omentaca by the Huaorani people	To prevent tooth decay.	Together with a small amount of safrole (0.74%), (*E*)-caryophyllene (**61**) (25.07%), α-humulene (10.48%), α-selinene (7.58%), β-selinene (6.08%), and α-phellandrene (5.37%) were the main constituents of the EO [92].	The EO showed strong antifungal activity against *Trichophyton rubrum* and *T. mentagrophytes* (both with an MIC of 500 µg/mL) and high antiradical activity (IC_50_ = 950 and 800 µg/mL in DPPH and ABTS assays, respectively) [92]. The alkaloidal extract exhibited stronger antiradical activity with IC_50_ values of 70 and 100 µg/mL in ABTS and DPPH assays, respectively [20].
Polygonaceae				
*Muehlenbeckia tamnifolia* (Kunth) Meisn.	Anku yuyu lutu yuyu	To treat kidney diseases and toothache, and as an anti-inflammatory agent in combination with other plants.	β-Sitosterol (**21**), lupeol (**37**), lupeol acetate (**74**), *cis*-*p*-coumaric acid (**75**), *trans*-*p*-coumaric acid (**76**), linoleic acid (**77**), (+)-catechin (**78**), afzelin (**79**), and quercitrin (**80**) (Figure 11) were isolated from the non-volatile fractions.	The hexane extract showed weak α-amylase inhibitory activity (IC_50_ = 625 µg/mL), while the other extracts and isolated compounds were inactive at the maximum dose tested. The hexane and methanol extracts exhibited strong inhibitory activity against α-glucosidase (IC_50_ = 48.22 and 19.22 µg/mL, respectively). Linoleic acid (**77**) (IC_50_ = 0.42 µM), afzelin (**78**) (IC_50_ = 3.56 µM), (+)-catechin (**79**) (IC_50_ = 5.50 µM), and quercitrin (**80**) (IC_50_ = 7.77 µM) were much stronger inhibitors than acarbose (377 µM) [93].
Proteaceae				
*Oreocallis grandiflora* (Lam.) R. Br.	Cucharillo in Loja and Zamora provinces, cucharilla in the Sierra region, gañal in Bolivar, and algil in Chimborazo province	Leaves and flowers are traditionally administered to treat liver diseases, ovary and uterus inflammation, and vaginal bleeding; moreover, they are used to prepare a digestive, diuretic, and hypoglycemic remedy.	Quercetin 3-*O*-β-glucuronide (**81**) and myricetin 3-*O*-β-glucuronide (**82**) (Figure 12) were isolated from a flower hydroalcoholic extract; in addition to these two flavonoids, quercetin 3-*O*-rutinoside (**83**) and isorhamnetin 3-*O*-rutinoside (**84**) (Figure 12) were detected in the leaf hydroalcoholic extract [94].	The leaf extract exhibited high antiradical and anti-inflammatory effects in the DPPH assay (IC_50_ = 6.69 ± 1.39 µg/mL) and the water-soluble tetrazolium salt WST-1 (IC_50_ = 4.08 ± 0.07 µg/mL) assay. Quercetin 3-*O*-β-glucuronide (**81**) showed an IC_50_ = 3.73 ± 0.11 µg/mL in a DPPH test [94]. The plant methanolic extract also showed remarkable inhibitory activity against α-glucosidase (IC_50_ = 2.8 ± 0.4 µg/mL) and α-amylase (IC_50_ = 161.5 ± 1.3 µg/mL) [19]. These findings justify the traditional medicinal uses of the plant, and suggest the potential use as a source of natural antioxidant, anti-inflammatory, and hypoglycemic products, and as a food supplement [19,94].
*Roupala montana* Aubl.	Palo de zorrilo	The infusion is used to treat nervous diseases.	Kaur-16-ene (**85**), linolenic acid (**86**), and α-tocopherol (**87**) (Figure 12) were isolated from a hexane extract; kaur-16-ene (**85**) (77.2%), kaur-15-ene (4.1%), phytol (**19**) (3.45%), (*E*)-nerolidol (**62**) (2.22%), and (5*E*,9*E*)-farnesyl acetone (1.2%) were identified as the main constituents of the EO that was steam distilled from dried plants using GC/MS and GC/FID [95].	
Rubiaceae				
*Alibertia sp.*	Suu	Anti-inflammatory, analgesic, and antimicrobial remedies.		Hexane extracts of the stems and leaves showed antiradical capacities with values of 58.6 ± 20.6 and 68.5 ± 15.1 µM TE/mg extract in ABTS and DPPH assays, respectively [20].
*Arcytophyllum thymifolium* (Ruiz & Pav.) Standl.	Canllye	Aerial parts are used in the Andean region to treat indigestion and colics.	The new coumarins **88**–**90**, the prenyloxy eriodictyol derivative **91**, and the iridoids **92** and **93**, in addition to known coumarins (**94**–**98**), flavonols (**99**–**101**), iridoids (**102**–**108**), and quinic acid derivatives **109** and **110** (Figure 13), were isolated from the plant [96].	The flavanone **91** showed potent α-glucosidase inhibitory effect (IC_50_ = 28.1 ± 2.6 µM), whereas asperulosidic acid (**102**) (IC_50_ = 69.4 ± 3.1 µM) and rhamnetin (**100**) (IC_50_ = 73.9 ± 5.9 µM) were active against α-amylase. Molecular modeling suggested the interaction of the flavanone **91** at the active site of α-glucosidase [96].
Sapotaceae				
*Mimusops coriacea* (A.DC.) Mig.	Manzana de mono (monkey apple)	Analgesic and anti-inflammatory remedies.	Two oils were extracted with hexane from the seeds of green and ripe fruits in a Soxhlet apparatus. Four fatty acids were identified using GC/MS in the saponifiable fraction, among which, 9-octadecanoic acid predominated (61.05%). The main constituents of the unsaponifiable fractions were squalene (81.26%) from green fruits and 3-oxo-urs-12-en-24-oic acid methyl ester (45.13%) from ripe fruits, respectively. Thirty-one compounds, including phenolic derivatives and triterpene saponins, were identified using LC-MS in the hydroalcoholic extracts of green and ripe fruits [97].	The green fruit extract showed the highest radical scavenging activity (IC_50_ = 4.99 ± 1.32 and 246.80 ± 6.87 µg/mL in DPPH and ABTS assays, respectively), followed by the ripe fruit extract with values of IC_50_ = 8.95 ± 1.24 and 250.30 ± 6.19 µg/mL, respectively. As for the anti-inflammatory activity, in a carrageenan-induced foot edema test, the extracts showed inhibition values higher than 50%, which were comparable with the reference drug indomethacin [97].
Siparunaceae				
*Siparuna echinata* (Kunth) A. DC.	Limoncillo, amapa, capitiú, or napanga	Fruit and leaf infusions are used to treat digestive disorders and rheumatisms.	α-Pinene (20.3–24.3%), β-pinene (21.7–22.7%), β-myrcene (11.3–14.8%), limonene (10.0-11.3%), *cis*-ocimene (8.1–8.5%), and *trans*-ocimene (8.4–8.9%) were identified in the volatile fraction. (+)-α-Pinene was enantiomerically pure, while the enantiomeric excess of (+)-β-pinene was 6.7%. The rare sesquiterpenoid diacetate sipaucin A (**111**) (Figure 13) was isolated from an EtOAc extract of dried leaves [98].	
*Siparuna muricata* (Ruiz & Pav.) A. DC.	Limoncillo	Antiacid	Four EOs, which were steam distilled from samples of the plant that were collected at four different localities of Ecuador, were compared using GC/MS and GC/FID. The main components of the sample that was collected at Chuquiribamba were guaiol (**34**) (14.61%), atractylone (13.21%), and *cis*-cadina-1(6),4-diene (13.31%); the main components of the sample collected in the Yangana sector were *cis*-cadina-1(6),4-diene (21.20%) and myrcene (16.57%); atractylone (17.70%), myrcene (12.70%), and germacrene B (12.18%) predominated in the specimen from the Celica sector; whereas, germacrene B (19.65%), myrcene (17.95%), and *cis*-cadina-1(6),4-diene (7.48%) were the main components of the EO from the plant collected in the Colaisaca sector. The enantiomeric excesses of β-pinene, limonene, δ-elemene, β-bourbonene, and *cis*-cadina-1(6),4-diene were determined using enantioselective GC. The altitude and the soil and leaf chemical elements influenced the chemical compositions of the EOs [99].	
Solanaceae				
*Datura stramonium* L.	Chamico or estramonio (stramonium)	To treat asthma, toothache, and Parkinson’s disease; it is also used as an antiparasitic and analgesic remedy.		The alkaloidal extract exhibited antiradical activity in DPPH (IC_50_ = 500 µg extract/mL) and ABTS (IC_50_ > 1 µg extract/mL) assays [20].
Tropaeolaceae				
*Tropaeolum tuberosum* Ruiz & Pav.	Mashua or cubios	To cure kidney and prostate disorders, liver pain, and skin eczemas.		The alkaloidal extract of leaves and stems showed an IC_50_ = 364.8 ± 18.9 µM TE/mg extract in an ABTS assay and an IC_50_ = 258.2 ± 38.0 µM TE/mg extract in a DPPH test [20].
Verbenaceae				
*Lippia citriodora* (Palau) Kunth	Cedrón	To flavor drinks, desserts, salads, and as a food seasoning agent; infusions of the leaves and flowers are used to treat respiratory and digestive problems.	Phytochemical screenings indicated the presence of tannins, polyphenols, triterpenes, and unsaturated sterols in the leaves, stems, and flowers; phenylpropanoids and catechins in the stems and flowers; alkaloids in the leaves and flowers; saponins in the leaves and stems; and coumarins and methylene ketones in the flowers.	The ethanolic extracts showed high antibacterial activity against *Escherichia coli*, *Staphylococcus aureus*, and *Pseudomonas aeruginosa*. The flower extract was moderately active against *S. aureus*. The flower and stem extracts showed high antifungal activity against *Candida albicans*, while the leaf extract was only moderately active. All the extracts were significantly lethal against *Artemia salina* after 24 h of exposure; the stem extract presented the highest activity (82.19 µg/mL), followed by the leaf extract (168.77 µg/mL) and the flower extract (172.76 µg/mL) [100].
Zingiberaceae				
*Curcuma longa* L.	Turmeric, curcuma, or safran	Turmeric extracts have shown hepatoprotective, antiparasitic, antifungal, and insect repellent activities; the plant is traditionally known for its fungicidal and bactericidal properties, and as a food seasoning agent.	Oxygenated compounds, such as ar-turmerone (45.5%) and α-turmerone (13.4%), and sesquiterpenes and monoterpenes, such as α-zingiberene (5.3%) and α-phellandrene (6.3%), were identified in the EO that was steam distilled from the rhizomes [101].	Low antioxidant activity was detected for the EO using DPPH and FRAP assays; instead, the EO exhibited good antibacterial activity against *Bacillus subtilis*, *Staphylococcus aureus*, and *Penicillium citrinum* [101].
*Renealmia thyrsoidea* (Ruiz & Pav.) Poepp. & Endlicher	Shiwanku muyu	A wide range of pharmacological properties, including antimalarial, antipyretic, analgesic, and anti-flu effects, and antidote activity against snake bites. A dye is extracted from the fruits, which is used in rituals by ethnic groups in the Amazon region.	Terpinolene (26.32%), α-phellandrene (17.16%), γ-terpinene (6.55%), β-pinene (5.97%), and *p*-cymol (4.70%) were the main constituents of the EO [102].	The EO exhibited strong antimicrobial activity against *Escherichia coli* and *Pseudomonas aeruginosa* (MIC = 0.35 mg/mL toward each bacterium) [102].
*Zingiber* officinale Roscoe	Ginger	To treat dyspeptic disorders and nausea.	Seventy-one compounds were identified in the EO isolated from rhizomes. The main components were citral (neral 9.1% and geranial 10.5%), α-zingiberene (17.4%), camphene (7.8%), (*E*,*E*)-α-farnesene (6.8%), and β-sesquiphellandrene (6.7%) [103].	The EO exhibited immunomodulatory and bronchodilatory effects. In vitro assays showed that the EO was active against the hydroxyl radical (IC_50_ = 0.0065 µg/mL), ABTS cation radical (IC_50_ = 3.94 µg/mL), oxygen radical (IC_50_ = 404.0 µg/mL) and DPPH radical (IC_50_ = 675 µg/mL); moreover, it had Fe(III)-chelating activity (IC_50_ = 0.822 µg/mL) and exhibited xanthine oxidase inhibitory activity (IC_50_ = 138.0 µg/mL). In vivo studies with *Saccharomyces cerevisiae* showed that the oil blocked the oxidation processes in yeast cells and significantly increased the antioxidant marker enzymes, superoxide dismutase (SOD), catalase (CAT), and glutathione peroxidase (GPx) in a dose-dependent manner. In addition, at a concentration of 1.6 mg/mL, the EO increased cellular viability under H_2_O_2_-induced oxidative stress. Thus, the antioxidant ability of ginger EO demonstrated its potential as a food additive and a phytotherapeutic remedy [103].

## Data Availability

Not applicable.
